# Monte-Carlo Modeling of the Central Carbon Metabolism of *Lactococcus lactis*: Insights into Metabolic Regulation

**DOI:** 10.1371/journal.pone.0106453

**Published:** 2014-09-30

**Authors:** Ettore Murabito, Malkhey Verma, Martijn Bekker, Domenico Bellomo, Hans V. Westerhoff, Bas Teusink, Ralf Steuer

**Affiliations:** 1 Manchester Institute of Biotechnology, School of Chemical Engineering and Analytical Sciences (CEAS), Manchester Centre for Integrative Systems Biology (MCISB), The University of Manchester, Manchester, United Kingdom; 2 Molecular Microbial Physiology, Swammerdam Institute for Life Sciences, University of Amsterdam, Amsterdam, The Netherlands; 3 Systems Bioinformatics IBIVU and Netherlands Institute for Systems Biology (NISB), VU University Amsterdam, Amsterdam, The Netherlands; 4 Synthetic Systems Biology, Swammerdam Institute for Life Sciences, University of Amsterdam, Amsterdam, The Netherlands; 5 Molecular Cell Physiology, FALW, VU University Amsterdam, Amsterdam, The Netherlands; 6 CzechGlobe - Global Change Research Center, Academy of Sciences of the Czech Republic, Brno, Czech Republic; 7 Humboldt-University Berlin, Institute for Theoretical Biology, Berlin, Germany; University of Georgia, United States of America

## Abstract

Metabolic pathways are complex dynamic systems whose response to perturbations and environmental challenges are governed by multiple interdependencies between enzyme properties, reactions rates, and substrate levels. Understanding the dynamics arising from such a network can be greatly enhanced by the construction of a computational model that embodies the properties of the respective system. Such models aim to incorporate mechanistic details of cellular interactions to mimic the temporal behavior of the biochemical reaction system and usually require substantial knowledge of kinetic parameters to allow meaningful conclusions. Several approaches have been suggested to overcome the severe data requirements of kinetic modeling, including the use of approximative kinetics and Monte-Carlo sampling of reaction parameters. In this work, we employ a probabilistic approach to study the response of a complex metabolic system, the central metabolism of the lactic acid bacterium *Lactococcus lactis*, subject to perturbations and brief periods of starvation. Supplementing existing methodologies, we show that it is possible to acquire a detailed understanding of the control properties of a corresponding metabolic pathway model that is directly based on experimental observations. In particular, we delineate the role of enzymatic regulation to maintain metabolic stability and metabolic recovery after periods of starvation. It is shown that the feedforward activation of the pyruvate kinase by fructose-1,6-bisphosphate qualitatively alters the bifurcation structure of the corresponding pathway model, indicating a crucial role of enzymatic regulation to prevent metabolic collapse for low external concentrations of glucose. We argue that similar probabilistic methodologies will help our understanding of dynamic properties of small-, medium- and large-scale metabolic networks models.

## Introduction

Lactic acid bacteria (LAB) are industrially important microorganisms used in the fermentation of milk, meat, and certain vegetables [1], [2]. LAB produce lactic acid as their main catabolic byproduct and are frequently used as starter cultures in various food-fermentation processes. LAB have a long tradition of safe use and, because the central metabolism of LAB has high activity, is an interesting target for metabolic engineering. A variety of metabolic models, detailed kinetic models as well as large-scale stoichiometric reconstructions, have been proposed, in particular for the best-studied LAB *Lactococcus lactis* [3]–[10]. Despite a relative wealth of data, however, many aspects of the fermentative metabolism of LAB are still insufficiently understood [2], [11]–[17]. Even comparatively small metabolic pathways, such as the glycolytic pathway of LAB, are highly interconnected not only via carbon metabolites, but also via redox (NADH) and energy carriers (ATP). Understanding the dynamic response arising from such a complex network of interactions is often not possible through heuristic reasoning alone, but requires the construction of detailed computational models. Such kinetic models of biochemical pathways are typically built in a bottom-up approach. First, the detailed enzyme-kinetic properties of each individual step are collected, having been sourced from either dedicated experiments or from literature research and databases. Subsequently, the model is constructed and revised until a faithful *in-silico* representation of the pathway is obtained. Despite considerable efforts, however, the construction of such models is still hampered by the extensive data requirements necessary to parametrize each enzymatic step. Several approaches have been suggested to alleviate this problem, including the use of approximative kinetics [18], [19] and Monte-Carlo sampling of reaction parameters [20], [21]. As one of the first applications of a Monte-Carlo sampling procedure accounting for the uncertainty in kinetic parameters, Wang et al. [22] employed statistical tools for the identification of the rate-controlling steps in a model of yeast glycolysis. Shortly afterwards, the formalism of generalized modeling [23] was extended to study the dynamic properties of models of metabolic pathways by using a general parametric representation of their Jacobian matrix [24], [25]. Later, these methods were adopted and modified by several other groups [26]–[30].

Building upon these methods, we aim to elucidate and understand the regulatory properties of the central metabolism of the lactic acid bacterium *Lactococcus lactis*. In particular, we seek to understand how allosteric regulation, together with its associated feedback and feedforward loops, influence the control exerted by the various enzymatic steps. Making only minimal assumptions about rate equations and kinetic parameters, and data from direct experimentation, we show that it is possible to study and elucidate the control properties of a metabolic pathway. In a second step, we investigate the dynamics of a corresponding kinetic pathway model in periods of starvation and show that allosteric control and regulatory interactions are crucial to maintaining metabolic viability in times of nutrient scarcity. Our probabilistic approach directly builds upon measured properties, such as the concentrations of metabolic intermediates and flux distributions, rather than enzyme-kinetic parameters, to constrain the possible dynamics of a metabolic pathway. We demonstrate that (i) the control coefficients of biochemical network models show intelligible patterns and trends that are accessible without detailed knowledge of enzyme-kinetic parameters; (ii) the regulatory structure of a biochemical network models has profound effects on the possible dynamics that are largely independent of specific kinetic parameters; and (iii) more specifically, that the topology of the regulation network is instrumental to ensure the stability of an observed state and to enable the patwhay to survive periods of starvation. We expect that our methodology will be of high utility to elucidate and understand the dynamic and regulatory properties that enable large-scale metabolic networks to function reliably in uncertain environments.

## Results/Discussion

### The Central Metabolism of *Lactococcus lactis*

**Figure 1:**
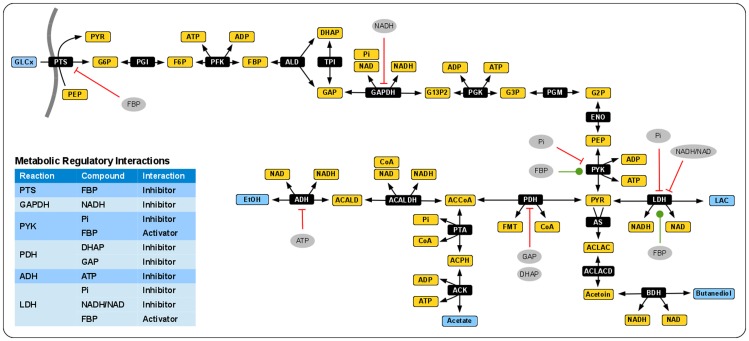
A pathway map of *L. lactis* central metabolism. The pathway involves 21 metabolic interconversions between 24 metabolites and includes three conserved moieties and two internal metabolites whose concentrations are assumed to be constant (FMT, CoA). External metabolites are indicated by light blue boxes. The inset provides an overview of the regulatory interactions. Abbreviations are defined in Text S2.

The starting point of our analysis is a stoichiometric representation of the central metabolism of *Lactococcus lactis*, defined here as the carbon and energy metabolism of this organism that generates most of its free-energy and C3 carbon precursors during fermentative growth. Drawing upon earlier kinetic models [3], [4], [7], [9], [15] and several available genome-scale reconstructions [5], [31], a set of enzymes involved in fermentative metabolism of *L. lactis* was selected. A graphical overview is shown in Figure 1. The metabolic network was chosen so as to describe the main glycolytic intermediates, the ATP regeneration cycle, and the dynamics of inorganic phosphate (Pi) and redox carriers (NAD/NADH). We neglect flux through the pentose phosphate pathway, since it accounts for less than 2% of glycolytic flux [32]. Main fermentation products are lactate (LAC), ethanol (EtOH), acetate and butanediol. Stoichiometric analysis reveals that the systems has three conserved moieties, ATP/ADP, NAD/NADH, as well as conservation of a phosphate group involving 11 metabolites. The concentrations of formate (FMT) and coenzyme A (CoA) are considered constant. The stoichiometry of the network allows for either LAC as the only fermentation product, or for equimolar amounts of butanediol and EtOH or acetate and EtOH as end products. The latter branch, fermentation to acetate and EtOH, results in the highest yield of ATP per glucose consumed.

Beyond the reaction stoichiometries, our model incorporates the currently known regulatory features found in *L. lactis* central metabolism. Fructose 1,6-bisphosphate (FBP) activates the formation of pyruvate (PYR) by the pyruvate kinase (PYK), activates the conversion from PYR to LAC by the lactate dehydrogenase (LDH), and inhibits the phosphotransferase system (PTS). A potentially important regulator is the concentration of free phosphate. In our model, the total pool of phosphate is constant, therefore the concentration of free inorganic phosphate (Pi) is an (inverse) measure of the amount of the glycolytic intermediates plus ATP. Further reactions that are under regulatory control include the pyruvate dehydrogenase (PDH), as well as the alcohol dehydrogenases (ADH). A complete list of all regulatory interactions is summarized in Figure 1 and given in Text S1. Following the workflow outlined above, a kinetic rate equation was assigned to each reaction step. In good agreement with earlier models [3], reaction rates were assumed to follow conventional Michaelis-Menten kinetics. Regulatory interactions were implemented as multiplicative factors to the rate equations, using Hill-type kinetics [33]. Detailed reaction equations are provided in Text S2. Subsequently, equilibrium constants were assigned to all steps, using values obtained by the group contribution method [34]. The metabolic state was based on values for extracellular fluxes and metabolites specifically measured for this study, supplemented with values from the literature. We consider a state with high external glucose (20 mM) and high-flux activity, see also Text S1.

### Probabilistic Analysis of Control Properties

**Figure 2:**
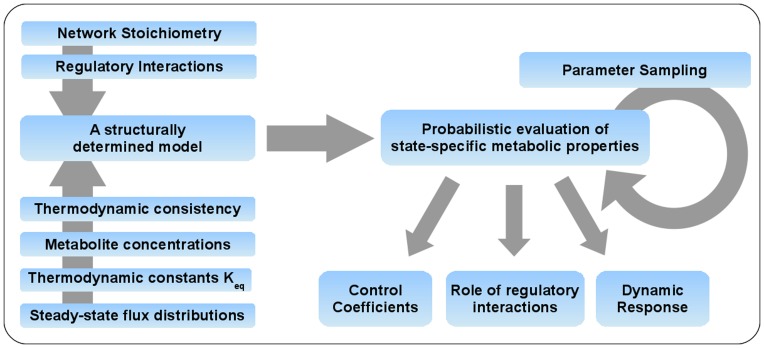
Probabilistic Modeling of *L. lactis*. The topological properties of the pathway, including the stoichiometry and all known regulatory interactions, are assembled. The analysis is then based on knowledge of a metabolic state of the system, as defined by a steady-state flux distribution and a set of associated thermodynamically consistent metabolite concentrations. Based on this information, the state-specific dynamic properties of the corresponding pathway model are evaluated. Of particular interest are control coefficients, the role of regulatory interactions, as well as the dynamic response to periods of starvation.

To quantify the extent to which a system property, such as a specific flux or metabolite concentration, is influenced by a specific enzymatic step, we make use of the concepts of Metabolic Control Analysis (MCA). The control coefficient 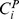 is defined as the relative change of a steady-state property 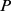 (the response of the system) divided by the small relative modulation of the activity of an enzyme 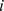 that caused that change [35], [36]. In the following, we mostly focus on the *flux control coefficients* 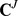, where the system properties 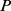 represent the fluxes within central metabolism. These control coefficients can only be evaluated for a fully characterized system, that is, their evaluation requires knowledge of the stoichiometry, as well as of all kinetic parameters or elasticity coefficients. Because neither all kinetic parameters nor all elasticity coefficients were known, we employed a Monte-Carlo scheme to iteratively sample enzyme-kinetic parameters, in particular Michaelis-Menten constants, to obtain insight into what we shall call the probabilistic distribution of control coefficients. To determine the respective intervals for the Monte-Carlo sampling of parameters, we make use of the fact that the control coefficients are essentially determined by the scaled elasticities, the logarithmic partial derivatives of the rate equations with respect to metabolic compounds. In a first approximation, these values are determined by the ratio of the Michaelis-Menten constants to their respective concentration [24], [28]. Motivated by the saturation properties of enzymatic rate equations, and making use of knowledge of the metabolic phenotype, we assume each Michaelis-Menten constant to be distributed around the concentration value of its associated metabolic compound. The width of the sampling interval is varied to test for robustness of the results; in the following all results are reported for a sampling interval spanning both, one order of magnitude above and below the respective concentration. After choosing all Michaelis constants, the forward velocity 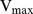 was adjusted so as to ensure that the reaction rate was not affected by the chosen constant. Applying this Monte-Carlo procedure, we obtain a distribution, rather than a single value, for each flux control coefficient. We emphasize that our approach ensures that each set of parameters is sampled such that it is consistent with the experimentally observed state. That is, each sampled set of paremeters indeed gives rise to the experimentally observed state. All subsequent evaluations are specific to this state. Prior to the calculation of control coefficients, the stability of the model is tested for each sampled set of parameters and only sets corresponding to a stable phenotype are retained (≈92% of all sampled instances). Our workflow is outlined in Figure 2.

**Figure 3:**
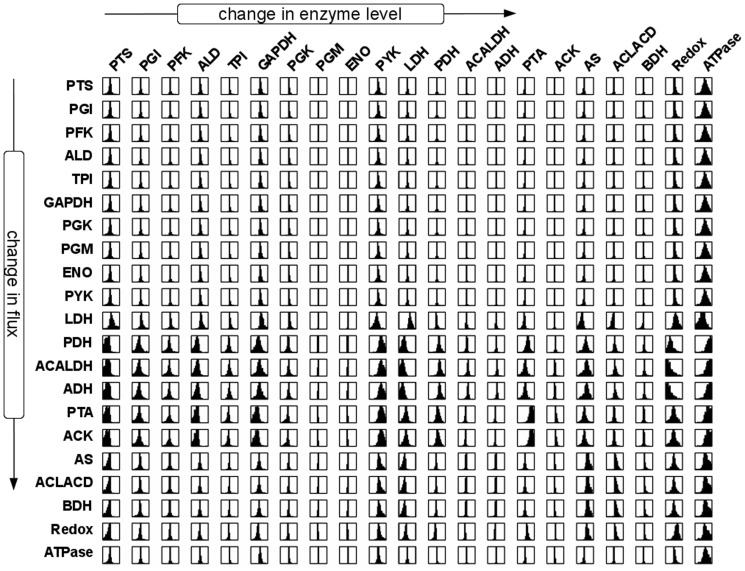
Probabilistic distribution of flux control coefficients. Shown is the distribution of the scaled flux control coefficients corresponding to the pathway model of *L. lactis* central metabolism given in Figure 1. Each plot corresponds to the interval [−1,1] on the abscissa. The diagram in the 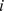
th column and on the 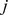
th row gives the distribution of the control coefficient quantifying the extent to which enzyme 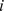 controls the flux through the reaction j. Each distribution provides information about the magnitude and uncertainty of one control coefficient. Narrow distributions indicate control coefficients that do not change appreciably due to parameter sampling, whereas broad distributions indicate that the precise value of the coefficient is more strongly dependent on parameter values. The corresponding sign distribution is shown in Figure 4.

**Figure 4:**
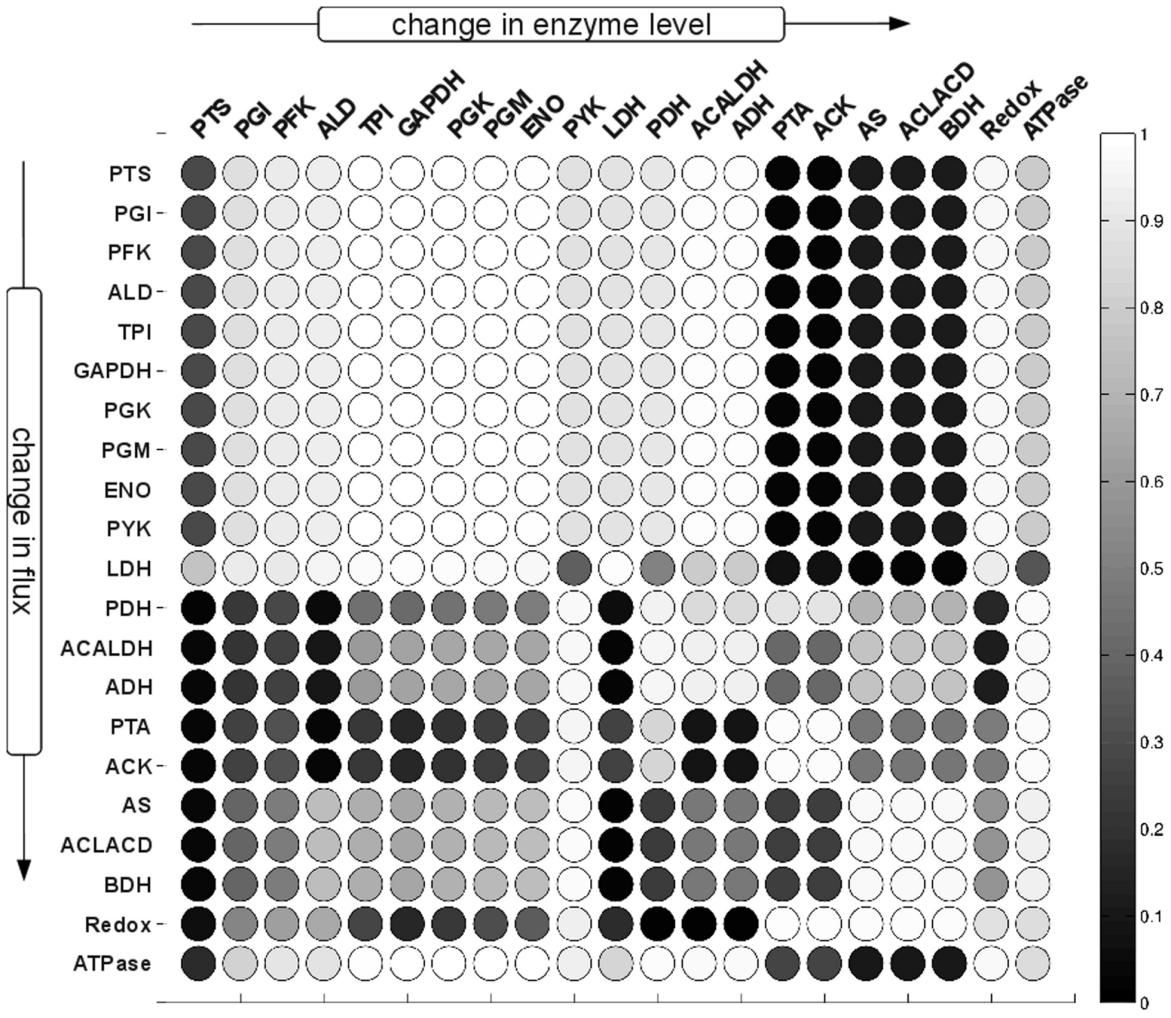
Probabilistic sign distribution of flux control coefficients. Grey-scale representation of the sign distribution of the flux control coefficient shown in Figure 3. The shade of the entry represents the percentage of the calculated control coefficients that are positive. Dark colors correspond to a distribution of flux control coefficients that lies predominantly on the negative semiaxis, whereas light colors indicate that the sampled control coefficients are predominantely positive. For example, for this metabolic phenotype, an increase of the enzyme PYK will for almost all sampled parameter values result in decreased flux through the LDH reaction as indicated by the dark circle in the row for LDH and the column for PFK.

The resulting distribution of flux control coefficients allows us to assess, qualitative and fairly independent of the precise values of the enzyme kinetic parameters, the potential effects of perturbations in enzyme levels on the system flux at the metabolic state. Most importantly, the distributions of the control coefficients were not arbitrarily. Rather, the results shown in Figure 3 exhibit strong patterns and, in accordance with earlier studies [37], indicate that qualitative knowledge of model properties can already result in well-constrained predictions, even when individual parameters are only poorly constrained. With respect to the interpretation of the distributions of scaled flux control coefficients, three properties are of particular interest: (i) The width of the distribution. Narrow distributions indicate control coefficients that do not change appreciably due to parameter sampling, whereas broad distributions indicate that the precise value of the coefficient is highly dependent on parameter values. (ii) The median of the distribution. For several reactions, the respective distributions are appreciably shifted away from zero towards either negative or positive values. (iii) The dominant sign of the control coefficients. Often, it is only of interest whether an increase in enzyme amount will result in an increase or decrease of a specific flux in the network, irrespective of the exact magnitude. To this end, Figure 4 provides the probabilistic sign distribution of flux control coefficients, grey-scale coded to indicate the percentage of sampled control coefficients that lie on the positive semi-axis.

### The Control of Flux

**Figure 5:**
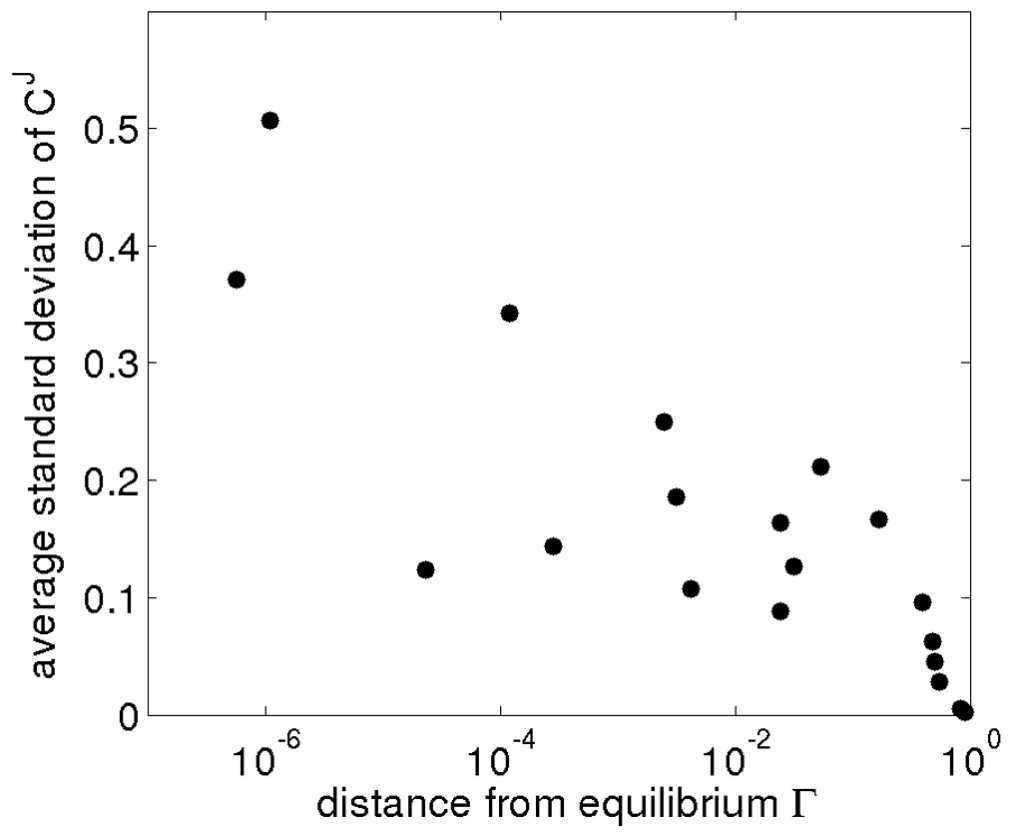
The width of the distribution of control coefficients correlates with distance from equilibrium. Shown is the average standard deviation of the sampled flux control distribution as a function of displacement 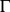 from equilibrium of the respective enzyme. Reactions close to equilibrium (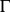 close to unity) typically have narrow distributions of flux control coefficients, centered at zero, indicating they can only exert little control over the flux through the system. Contrary, reactions far from equilibrium (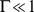) exhibit broad distributions, indicating a potential, but no necessity, for high control coefficients. For definitions see Materials and Methods.

Figures 3 and Figure 4 show the resultant distributions of control coefficient based on the sampling procedure described above. All sampled sets of parameters share the common property that they are consistent with the experimentally observed metabolic state of the pathway. However, other than that, we have no knowledge as to whether an actual control coefficient is close to the median value of the respective distribution, or whether evolutionary pressure has selected for a value at the extreme fringe of this distribution. Clearly, for each control coefficient, both scenarios are possible. To provide a meaningful interpretion of these results, we therefore proceed in two steps. First, we asses to what extend the observed distributions correspond to our intuitive assumptions about control within the pathway. Overall, the pattern of flux control coefficients shown in Figures 3 and 4 are indeed in good agreement with conventional assumptions about the distribution of control within the pathway. For example, the glycolytic enzymes (PGI to ENO) predominantly show positive control over the glycolytic flux, with more than 80% of the sampled control coefficients having positive values. Reactions close to equilibrium, such as PGM or ENO, typically also have narrow distributions around zero, indicating that they typically exert little control on the fluxes through the system, irrespective of precise parameter values. Indeed, the average standard deviation of the scaled control coefficients correlates with the distance from equilibrium of the respective reaction, as shown in Figure 5, and in accordance with theoretical considerations [38]. Other straightforward results include the predominantly negative control the LDH exerts upon the competing branches involved in the production of ethanol, acetate and butanediol. However, other properties of the distributions shown in Figure 3 and Figure 4 are less straightforward to explain. For example, the predominantely negative control that the enzymes involved in acetate production (PTA and ACK) exert upon the glycolytic flux. Although increasing activity of PTA and ACK might be expected to pull more flux through the upper part of glycolysis, the flux control coefficients indicate that this is, for the vast majority of parameters, not the case. Rather, increasing the activity of the acetate branch prevents the regeneration of NAD required by the glyceraldehyde-3-phosphate-dehydrogenase (GAPDH), consequently slowing down the glycolytic pathway.

We emphasize that the probabilistic distributions shown in Figures 3 and 4 depict control properties of the pathway model for values of kinetic parameters that are consistent with the experimentally observed state, but are otherwise random. Clearly, the peaks of these distributions do not necessarily correspond to the actual control profile of the organism. Selection pressure during evolution might have easily led to parameters on the fringes or even outside of the range in which we sampled, precisely because the organism's requirements were different from what arises as ‘typical’ from random sampling. We therefore must compare the distributions shown in Figures 3 and Figure 4 with known and empirically obtained control coefficients in *L. lactis* [39]–[42]. In particular, control coefficients that take values far from the median value of the respective distribution might point to additional evolutionary pressure, or errors in the topology of the pathway, and therefore provide valuable information for further analysis.

Using a series of mutants with altered GAPDH activity, Solem et al. (2003) [40] determined that changes in GAPDH activity had virtually no effect on glycolytic flux in growing as well as nongrowing cells. Likewise, in a series of studies, the enzymes PFK, PYK, and LDH, encoded together on the *las* operon, were shown to have no significant control on the glycolytic flux in exponentially growing cells [39], [41]. These findings are in agreement with the rather narrow distributions, centered at zero, of the respective probabilistic control coefficients. Furthermore, Koebmann et al. (2005) [41] showed that the enzyme LDH has a strong negative control over the flux to mixed acids and formate formation, whereas PYK has a strong negative control over these fluxes – again in agreement with the respective distributions. PFK was found to have no control on either acetate or lactate flux, again corresponding to the respective distribution. An interesting case is the control of ATP consuming-processes outside of the pathway (ATPase) on the glycolytic flux. The respective probabilistic distribution indicates a large potential for control, as manifested by the very broad distribution of the corresponding flux control coefficients. Such a high level of control by ATP consuming reactions outside of the pathway was indeed reported recently [17], and a similar high control of demand for ATP on glycolytic flux has been observed for *E. coli* [43]. From the introduction of an uncoupled ATPase activity, however, Koebmann et al. (2002) [39] concluded that the flux control by ATP demanding processes was close to zero over a range of ATP/ADP ratios. Both scenarios are consistent with the broad distribution shown in Figure 3. We emphasize that in the following, despite the good agreement with experimental data, we do not interpret the probabilistic distributions as a likelihood for the actual control coefficients. Rather the distributions indicate ranges of control coefficients that are typical for the metabolic state, given our sampling procedure, whereas deviations provide a highly valuable starting point for further analysis. We note, however, that as yet most empirically determined control coefficients available for *L. lactis* do seem to be consistent with the bulk of the respective distribution.

### Control of Glycolytic Flux by PTS

**Figure 6:**
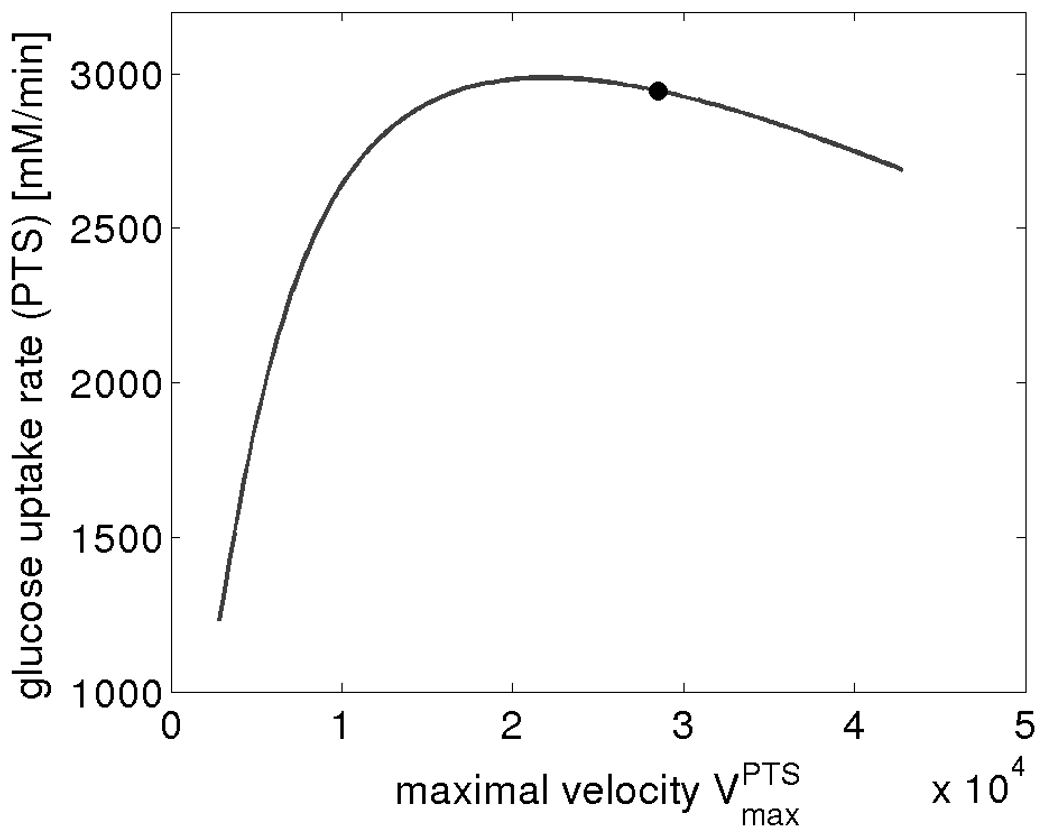
Dependency of the glycolytic flux on the maximal activity of the glucose transporter (PTS). Shown is the glucose uptake as a function of 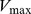, evaluated using a kinetic model with a representative set of the sampled parameters. The black dot indicates the reference state.

As a rather counter-intuitive finding, the distribution of control coefficients indicates that for most sets of parameters the PTS would exert a negative control on flux through the pathway. This finding is in contrast to our expectation that an increase in PTS activity results in higher glucose uptake, hence an increased flux through glycolysis. These results emphasize that the control coefficients are indeed systemic properties of the pathway and may exhibit non-intuitive patterns, depending on the specific metabolic phenotype and details of the pathway stoichiometry and regulation. To verify that the non-intuitive distribution of the influence of the glucose transport upon the glycolytic flux is indeed a true feature of the pathway model, we varied the maximal velocity (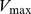) of the PTS using an explicit kinetic model. The resulting flux is shown in Figure 6 for a specific set of reference parameters. Repeating the analysis for a large number of sampled parameters yielded similar results. At the specified metabolic state, the slope of the curve representing glucose uptake as a function of 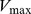 is almost always negative. The universality of the negative slope indicates that the control profile of the network model is indeed strongly dependent on the specific metabolic phenotype at which the probabilistic control profile is evaluated. That is, the experimentally observed metabolic state itself shapes the resultant distribution of control coefficients. To verify this assertion, we evaluated the control profile of a second metabolic state, corresponding to a situation with low external glucose. The results are provided and discussed in Text S2. In this case, the control of the PTS system on glycolytic flux is predominantly positive.

### The Impact of Metabolic Regulation

Up to this point, the system had been evaluated in the presence of metabolic regulation additional to substrate/product effects. Each instance of sampled parameters included randomly selected values for all regulatory interactions. To delineate the impact of metabolic regulation, we subsequently removed all regulatory interactions and repeated the analysis to pinpoint specific differences between the regulated and unregulated network model. In particular, our re-scaling and sampling procedure allows us to analyze both systems at an identical steady state and therefore enables a direct comparison between both scenarios. We note that this approach is different to a simple change of parameters within a kinetic model, for example by decreasing the value of a certain feedback parameter. While such a change would indeed modify the feedback properties, it would also alter the metabolic state of the pathway and thereby complicate a direct comparison of systemic properties between both scenarios. In contrast, here, we assume that the metabolic phenotype, the set of concentrations and fluxes, has evolved to satisfy the particular functional requirements of the cell. By removing regulatory interactions while keeping the metabolic state unchanged, we ask the question how this evolved state would typically behave in the absence of metabolic regulation, whilst fulfilling the same metabolic function. As is demonstrated below, this approach allows us to perform a detailed assessment of the role of individual regulatory interactions.

**Figure 7:**
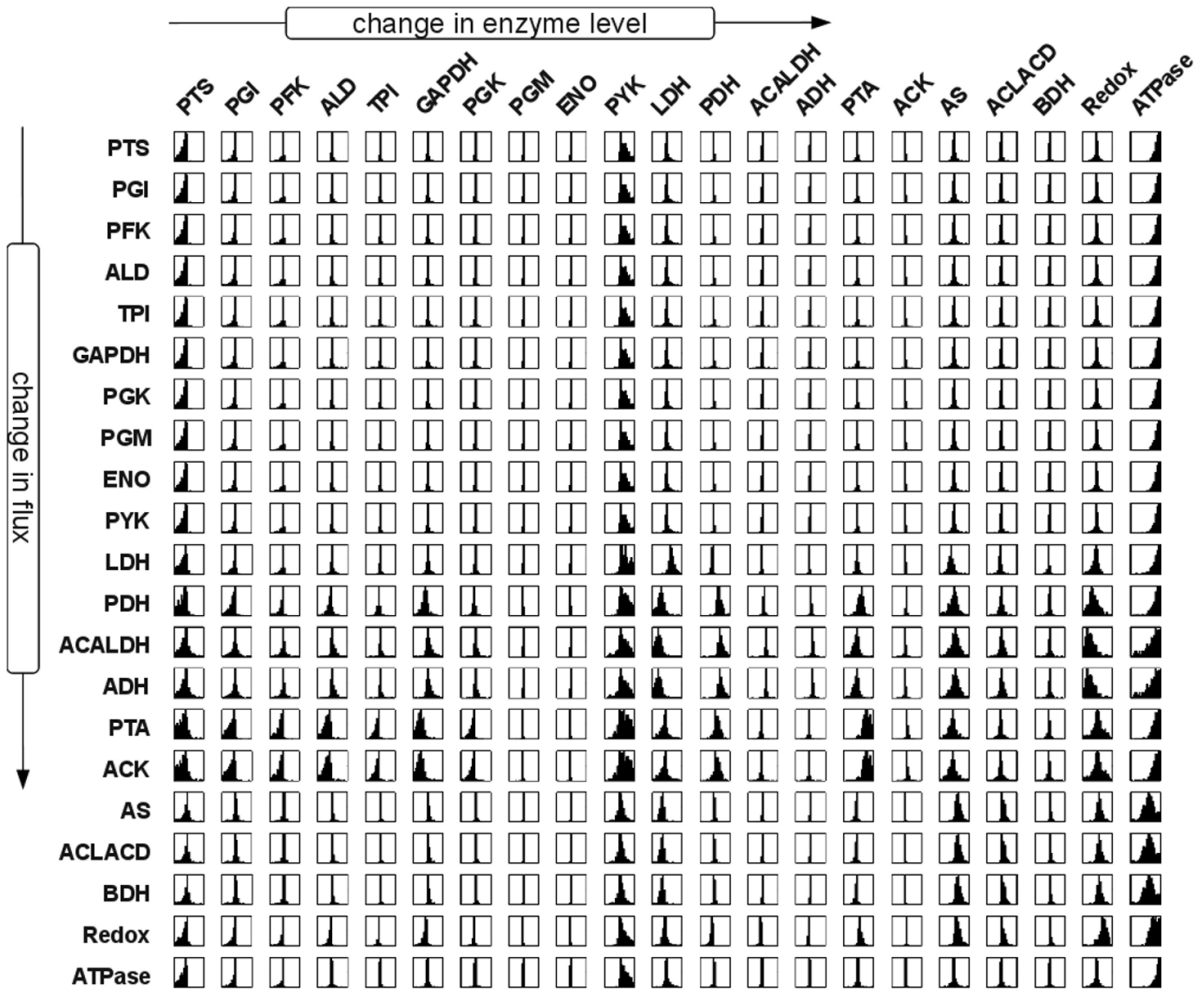
Probabilistic distribution of flux control coefficients in the absence of metabolic regulation. Same as Figure 3 except for the absence of metabolic regulation. Any diagram refers to the control of one flux (i.e. through the step indicated to the left of the row) by one enzyme (i.e. the enzyme indicated above each diagram corresponds to the interval [-1,1] on the abscissa.

**Figure 8:**
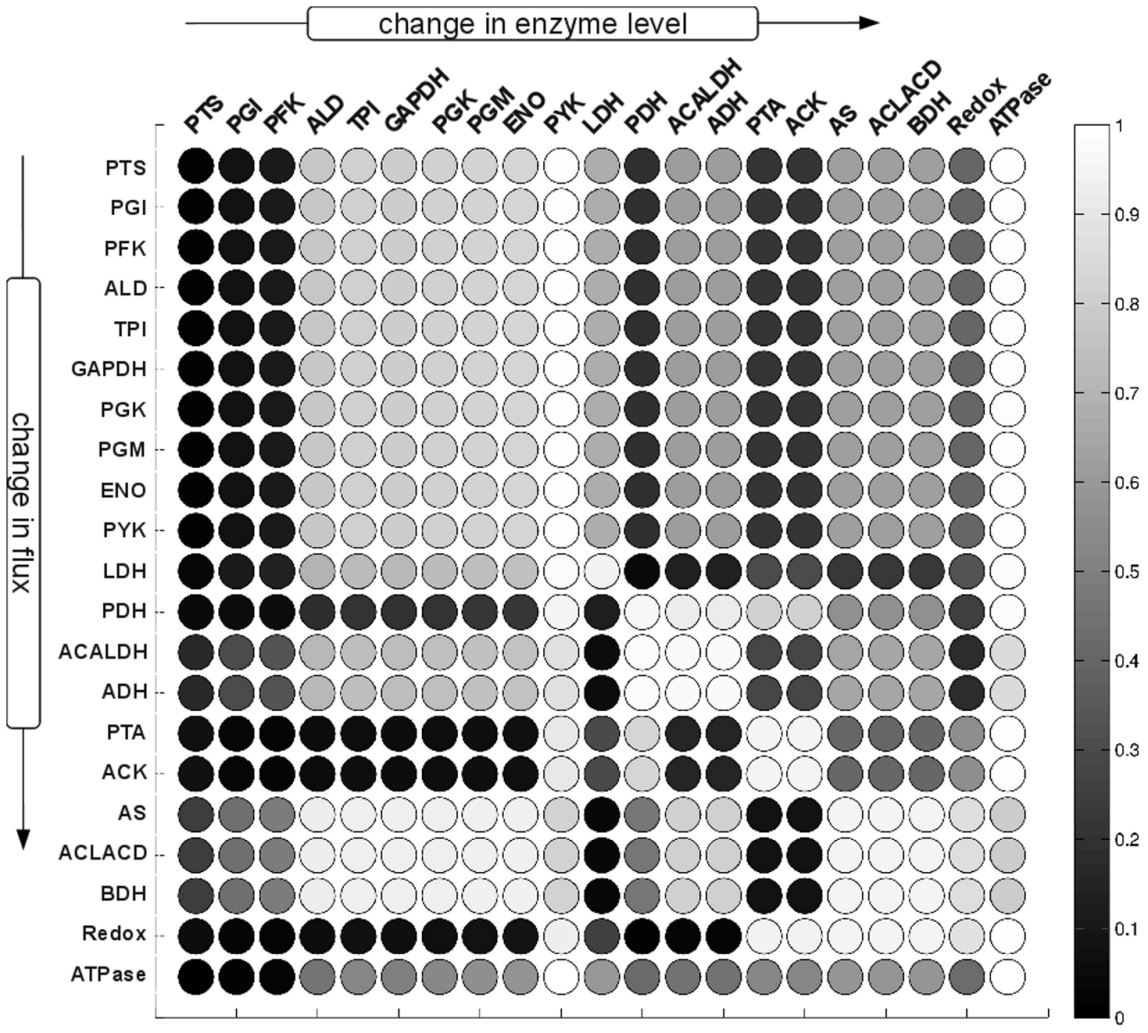
Probabilistic sign distribution of flux control coefficients in the absence of regulation. The shade of the entry represents the percentage of the calculated control coefficients that are positive. Dark colors correspond to a distribution of flux control coefficients that lies predominantly on the negative semiaxis, whereas light colors indicate that the sampled control coefficients are predominantly positive.

Repeating the Monte-Carlo sampling of kinetic parameters in the absence of regulatory interactions, we first note that the percentage of stable models, as evaluated by an analysis of the largest real part of the eigenvalues of the Jacobian, drops to about 83% of sampled instances, as compared to 92% for the regulated system considered above. The analysis was repeated for the second metabolic state, characterised by low external glucose, in the Text S2, showing that a similar drop in average stability is again associated with the absence of regulatory interactions. Figure 7 shows the probabilistic distribution of the scaled flux control coefficients in the absence of metabolic regulation, the corresponding sign distribution is shown in Figure 8. The absence of regulation affects the control profile in terms of the dominant sign of the control coefficients, as well as the amplitude and width of their distribution. A comparison between Figures 3 and 7 shows, for example, that the control of PGI and PFK over the glycolytic flux is predominantly positive in the regulated system, but almost always negative in the unregulated systems. Differences are also recorded for the regulation of the glycolytic flux by AS, ACLACD and BDH, which is negative in the regulated case and evenly distributed between negative and positive values in the absence of regulation. A strong difference between the probabilistic control profiles is also observed for the ATPase, representing general ATP demand outside of the pathway. In the presence of regulation, the distribution of the control coefficients of the ATPase on the glycolytic flux is rather broad, indicating a high potential for control, and symmetrically distributed around zero. In contrast, in the absence of regulation, the distribution is shifted to higher values, with a median value close to unity. Consequently, in the absence of metabolic regulation, the pathway model exhibits a strong sensitivity with respect to ATP demand for almost all possible sets of parameters. Similar differences are observed for the possible control of PYK on the glycolytic flux. The respective distribution is significantly narrower in the presence of metabolic regulation, indicating less potential for control but an increased robustness of the flux with respect to perturbations in enzyme levels. Similar differences in widths and signs were observed for the second metabolic state as discussed in Text S2.

### Starvation and Dynamic Recovery

**Figure 9:**
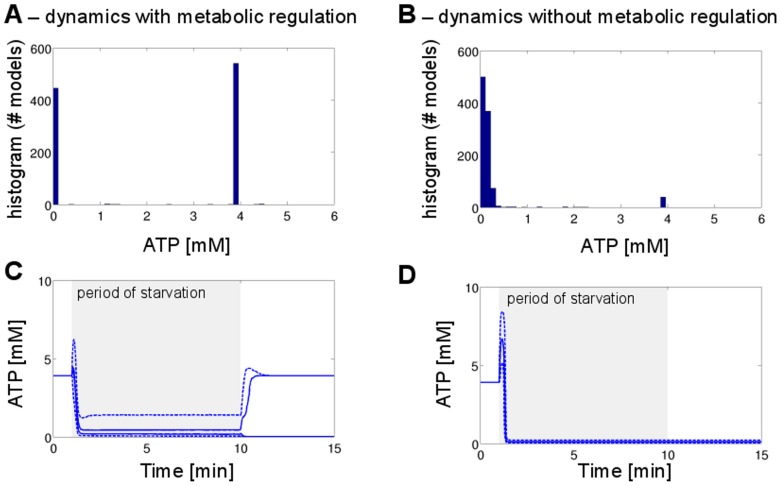
Metabolic recovery after periods of starvation. Starting at the defined metabolic state, external glucose is lowered from 20 mM to 0.1 mM at time t = 1 min, mimicking a brief period of starvation. At time t = 10 min external glucose is restored to its orginal value. The upper panel shows a histogram of intracellular ATP after a recovery period at time t = 100 min. For the regulated system (A), approximately 54% of all models recover to the initial metabolic state (542 of 1000 instances tested), whereas in the absence of regulation the probability of recovery is below 5% (39 of 1000 instances tested). The lower panel shows the median of the time-course, with .25 and 0.75 quantiles included as dashed lines. For the regulated system, the time-course is split between recovering and non-recovering instances.

To investigate the impact of metabolic regulation on the dynamic properties of the system, we have to go beyond the steady-state control coefficients and consider explicit time-courses of the system. In particular, we are interested in the dynamic response to periods of starvation and in the subsequent recovery when external nutrients are replenished. To this end, we set up an ensemble of models, each at the steady-state defined by the metabolic state considered above. Subsequently, we ran explicit numeric simulations for 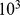 instances of sampled parameters in the presence and absence of regulatory interactions. After a period of 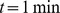, the external glucose was lowered from its original value of [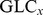]  =  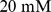 to a value of [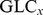]  =  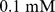. The pathway model then adapted to the new conditions and converged to a new metabolic state. At a simulation time of 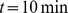, the external glucose was restored to its original value [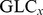]  =  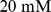. As expected, and shown in Figure 9, withdrawal of external glucose resulted in a quick drop of intracellular metabolites. Typical time-courses of selected model instances are provided in the Text S2. Due to the autocatalytic nature of the glycolytic pathway, the concentration of ATP typically first exhibits a slight increase, corresponding to the cessation of ATP utilization in the upper part of glycolysis. Subsequently, the concentration of ATP, and likewise of other metabolites, drops to a new steady-state, characterized by a significantly decreased ATP concentration. The average value of ATP concentration during starvation is 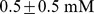 for regulated systems versus 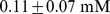 in the absence of regulation.

**Figure 10:**
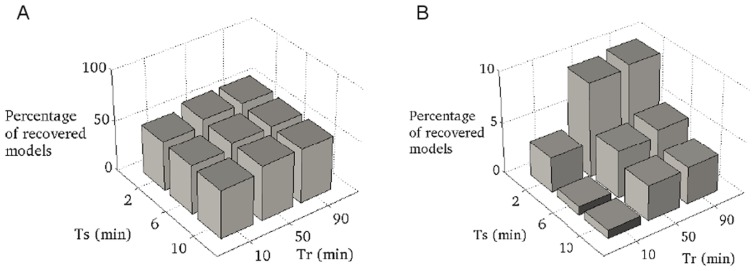
Recovery as a function of starvation time. Shown is the percentage of model instances that recovered to the original metabolic state after a starvation time 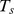 and a recovery time 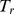 in the presence (left panel) and absence (right panel) of regulation. We emphasise the different scales on the z-axis on both panels. In the presence of regulation, the probability to recover does not appreciably depend on length of starvation and recovery time. In the absence of regulation a longer starvation time decreases the probability to recover.

Both scenarios exhibit drastically different behavior after external glucose is restored to its original value. For the regulated system, the distribution of ATP concentration after a suitable recovery time is clearly bimodal (Figure 9A). Approximately 54% of all models recover to the original metabolic state (542 of 1000 instances tested) and the ATP level reaches its original value (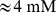). In the absence of regulation, the probability of the system to recover drops significantly. More than 95% of the simulations correspond to a failure in restoring the original concentration of ATP. The probability of the system to have recovered from starvation also depends on the duration of the starvation (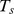) and recovery time (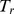). Figure 10 shows the percentage of recovered systems as a function of 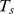 and 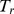 for both scenarios. In the presence of regulation, the probability to recover does not seem to depend appreciably on the duration of the starvation and recovery time. In the absence of regulation, however, a longer starvation time strongly decreases the probability of recovery. In addition, unregulated systems tend to recover more slowly, as also observed in the individual time-courses provided in the Text S2. These results are in agreement with the previous observation that regulatory interaction can accelerate response times of simple biological network motifs [44], [45].

### Screening Individual Regulation Mechanisms

To investigate the role of regulation in the response to periods of starvation and the subsequent metabolic recovery in more detail, we tested the response of the system in the presence of individual regulation mechanisms. To do so, we performed the aforementioned parameter sampling on models of the pathway endowed with only a single regulatory interaction. The percentage of models that recover after a period of starvation strongly depends on the specific regulatory interaction. Table 1 lists the percentage of recovering models for each individual regulation mechanism, along with the percentage of stable Jacobians for the respective system. Most of the regulatory mechanisms do not have any appreciable effect on the ability of the system to recover from starvation. Three of the ten tested regulation mechanisms, however, result in a significantly increased probability to recover after a period of starvation. The strongest increase is observed for FBP as an activator of PYK, followed by free inorganic phosphate (Pi) as an inhibitor of the same reaction. A slight, but significant, increase is also observed for FBP as an inhibitor of PTS. The results are in good agreement with previous work on metabolic stability using minimal pathway models [2], such as the study of Voit et al. [15] who investigate the feedforward activation system in *L. lactis* using a six variable model with generalized mass action kinetics. Likewise, recent work on regulation of PEP utilization in *E. coli* also emphasized the importance of FBP as a regulator allowing rapid adaptation to changing environmental conditions [46].

**Table 1:** Percentage of recovering systems in the presence of individual regulation mechanisms.

Reaction	Regulator	Type	Percentage Stable States	Percentage Recovering Systems
PTS	FBP	Inhibitor	87%	11%
GAPDH	NADH	Inhibitor	81%	4%
PYK	Pi	Inhibitor	97%	26%
PYK	FBP	Activator	87%	51%
PDH	DHAP	Inhibitor	83%	4%
PDH	GAP	Inhibitor	83%	3%
ADH	ATP	Inhibitor	82%	3%
LDH	Pi	Inhibitor	84%	2%
LDH	FBP	Activator	83%	2%
LDH	NADH/NAD	Inhibitor	83%	3%

The table gives the percentage of stable instances of the Jacobian in the presence of a single regulation mechanisms (83% in the absence of regulation) and the probability of metabolic recovery (4% in the absence of any regulation). The percentage of the recovering systems is always relative to the instances of stable systems tested.

### FBP as a regulator of PYK and PTS

**Figure 11:**
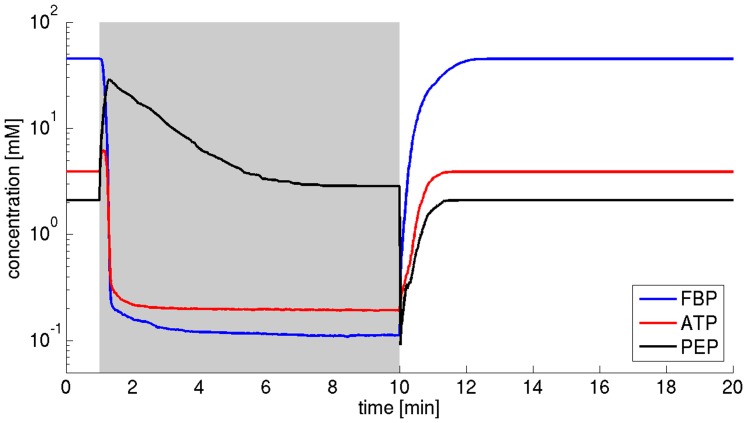
Time-courses of intermediate metabolites. The median of the concentration of FBP, ATP, and PEP following a withdrawal of external glucose at 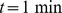 in models that include activation of PYK by FBP is shown. After withdrawal of glucose, the concentration of PEP quickly rises and attains a new steady state. With the restoration of external glucose at 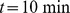, PEP undergoes a quick drop, fuelling glucose uptake and subsequent production of ATP. The corresponding figure for systems that lack regulatory interactions but are nonetheless able to recover from periods of starvation is discussed in the Text S2.

The importance of allosteric regulation of PYK by FBP for metabolic functioning, in particular for the levels of ATP and glycolytic intermediates, is widely recognized and the regulatory mechanisms is present in almost all glycolytic pathways [2], [46]. Our results suggest that for *L. lactis* at the metabolic state studied here, a major role of the activation of PYK by FBP is to prevent an irreversible metabolic collapse during brief times of starvation. Indeed, as argued previously [2], [15], the mechanism introduces a safety valve for the utilization of PEP. In the absence of regulation, and after the withdrawal of glucose, PEP is continually converted to pyruvate and short-lived ATP until all resources are depleted. However, in the presence of regulation, a decrease of FBP concomitantly decreases the utilization of PEP by PYK. Since no external glucose is present, utilization of PEP by PTS is likewise diminished. Hence a substantial amount of PEP is retained in the system, which allows glucose uptake and regeneration of ATP as soon as external glucose is restored to its pre-starvation levels. Figure 11 shows the time-course of the median of the concentrations of FBP, ATP, and PEP following the withdrawal of external glucose at 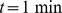 for models that include the activation of PYK by FBP. As expected, after withdrawal of glucose, the concentration of PEP quickly rises and attains a new steady state – interestingly with a median concentration similar to the value before starvation. With the restoration of external glucose at 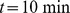, PEP undergoes a quick drop, fuelling the uptake of glucose, and the subsequent production of ATP. The time-course shown in Figure 11 is in strong contrast to the situation in the absence of feedforward regulation, even when selecting for parameters that likewise allow for metabolic recovery. The corresponding plot and its discussion is provided the Text S2. We note that a similar reasoning might also hold in the absence of a PTS. In this case, the substrate ATP is required for the hexokinase and PFK in upper glycolysis, which is provided by the PYK reaction. The functional role of the FBP-mediated negative feedback on the PTS is more difficult to delineate and has received only little attention so far [2]. While the feedback also contributes to metabolic stability, its quantitative effect is rather small. Functionally, the regulation constitutes a traditional negative feedback loop, providing a mechanism for pathway homeostasis.

### Metabolic Collapse and Hysteresis

**Figure 12:**
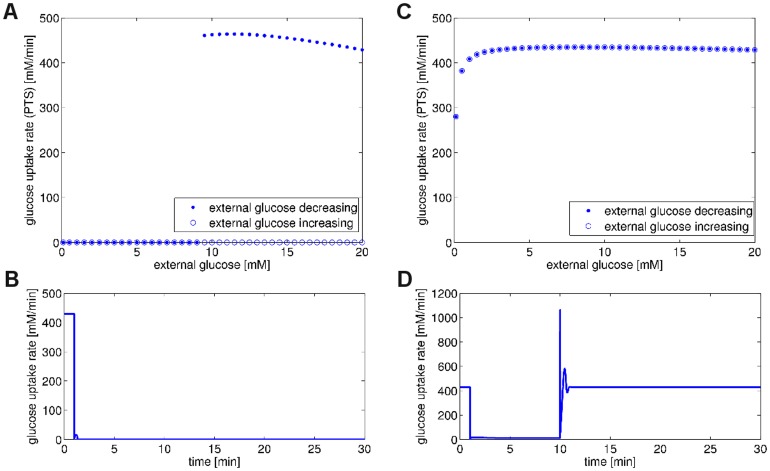
Bistability and hysteresis with respect to external glucose. Shown is a non-recovering system in the absence of regulation (A,B) and a recovering system in the presence of regulation (C,D). In the upper panels (A,C), the concentration of external glucose was varied between the initial level of 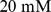 to a lower value of 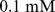 and back. The lower panels (B,D) show a corresponding time-course of the rate of glucose uptake. The original level of external glucose, 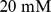, was lowered to 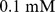 within the time interval t = 1 min to 10 min.

As demonstrated, the regulatory structure of the glycolytic pathway is crucial to allow for metabolic recovery after periods of starvation. This raises the question about the detailed dynamic mechanisms through which recovery is achieved. By definition, systems that fail to recover after the external glucose has been restored to its original value must at least possess one additional stable state for the respective concentration of external glucose, that is, the systems must be at least bistable. To test for this hypothesis, we varied the concentration of external glucose and progressively decreased the concentration from the initial 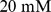 to 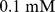. For each level of external glucose, the respective systems were allowed to relax to a new steady state. Subsequently, the external glucose was progressively increased again, back to the original value of 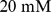. Two typical examples are shown in Figure 12. Indeed, bistability and hysteresis was observed for all non-recovering systems. For recovering systems, in almost all cases (approximately 99%) no hysteresis was observed. A small subset of recovering systems (approximately 1%), however, also exhibited hysteresis. In this case the hysteresis loop is usually fully contained within the considered interval of external glucose and the system is monostable for an external glucose concentration of 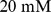. In very rare cases, the system also returned to its original state, despite hysteresis and the existence of a second stable state with low metabolic activity. In these cases, the sudden withdrawal and reestablishment of external glucose may induce dynamic transients that allow the system to leave the lower state. Figure 12 shows a non-recovering system in the absence of regulation (panels A and B), as well as a recovering system in the presence of regulation (panels C and D). We note that hysteresis was tested numerically and therefore the existence of further metabolic states cannot be excluded. However, the numeric results obtained from the tested 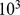 instances clearly showed that recovery was predominantely due to the monostability of the steady state for the respective concentration of the external glucose. Furthermore, the absence of bistability was clearly linked to the regulatory structure of the system, almost irrespective of the precise paramater values. Our results therefore imply that the regulatory structure indeed has a structural influence on the possible bifurcations of the pathway independent of a specific fine-tuned set of parameters.

## Conclusions

Metabolic regulation is a crucial aspect of cellular function. While significant progress has been made on the stoichiometric properties of large-scale metabolic systems, the organizing principles of metabolic regulation that enable stability and rapid adaptation are still insufficiently understood [15], [29], [38], [47]. In this work, we have presented a Monte-Carlo approach to study the regulatory feedback topology of the homo-fermentative LAB strain *Lactococcus lactis*. In particular, we have used knowledge of an experimentally acquired metabolic state to evaluate typical dynamic properties of a corresponding pathway model. To this end, our first aim was to demonstrate that the distributions of control coefficients obtained from conditional Monte-Carlo sampling is highly structured. Based on a sampling scheme, such that all considered sets of parameters are in accordance with the experimentally observed state, the resulting patterns of control coefficients were consistent with many well-known control control principles of the pathway.

Given these findings, two aspects of our study must be emphasized: First, we did not conduct a straightforward Monte-Carlo study such that the kinetic parameters were drawn from a random distribution. Such an approach would likely result in a plethora of different metabolic states – most of which are totally irrelevant for the functioning of the system under any condition. Rather, our approach is based on the assertion that evolution and selective pressure can easily restrict the metabolic state, and hence the parameters, to values that are beneficial for the organism's survivial, even if these parameter sets represent only an exceedingly small portion within parameters. Therefore, we restrict the sampling to an evolved metabolic state, such that all sampled parameters are consistent with this experimentally observed state. The underlying assumption is that the primary function of a metabolic pathway is embodied in its flux and, to a lesser extend, in the set of metabolite concentrations. It is these values that are the targets of selective pressure, not the kinetic parameters *per se*. We therefore seek to study how the dynamic properties of the pathway differ for different potential sets of kinetic parameters that are equally suited to support the observed steady-state flux. Such an approach allows us to straightforwardly compare the distributions of dynamic properties in the presence and absence of a specific metabolic feedback.

The second aspect of our study relates to the question: How relevant are such distributions of control properties, given that any actual system only attains a single set of parameters. To what extent is, therefore, average behavior an indicator for actual behavior, given that the actual set of parameters may as well be located on the extreme fringes of the distribution. While we reject any interpretation of average behavior as a likelihood for actual behavior, we claim that the distributions obtained using our computational procedure indeed have functional, as well as explanatory, relevance. The *explanatory* relevance lies in the fact that probabilistic control profiles allow us to evaluate to what extent experimental findings are rooted in the topological structure of the pathway, rather than in fine-tuning of Michaelis-Menten parameters. Specifically, if almost all sampled parameters attain a value within a certain narrow range, and the empirically observed value is in good agreement with these values, then we face no further explanatory challenge: The empirical value simply corresponds to what we expect as typical behavior, given no further knowledge about additional contraints the system is subject to. On the other hand, an empirically obtained control coefficient that is located at the extreme fringe of the respective distribution provides valuable information for further analysis and points to additional constraints or requirements the pathway is subject to. The *functional* relevance of our probabilistic evaluation is then given by the fact that control properties that are already inherent in the topological structure allow for an evolutionary adaptation of parameters with respect to other objectives. Specifically, it seems favorable, within the course of evolution, to adopt a network topology that exhibits a certain desired behavior, such as recovery after periods of starvation, for a broad range of parameter values. These parameters can then be further fine-tuned according to other objectives, without impeding the core functionality of the pathway.

In this sense, we argue that our approach has led to increased understanding of principles of metabolic regulation in *Lactococcus lactis*: We compared the typical response of the pathway with respect to short periods of starvation in the presence and absence of metabolic regulation. It was shown that the regulatory interactions, irrespective of the particular parameter values, result in qualitative differences in the dynamics. Our results therefore shows that the topology of the regulation alone is, to a large extent, already sufficient to ensure dynamic stability and recovery of the pathway. While fine-tuning of parameters may achieve a similar increase in stability, an appropriate regulatory structure dramatically increases the set of accessible parameters space and therefore opens the possibility to optimize Michaelis-Menten parameters for other secondary objectives, such as the trade-off between affinity and catalytic rate which are not considered here. The main result presented in this study is therefore that the regulatory architecture of the *Lactococcus lactis* central metabolism induces *qualitative* changes in the probabilistic control profile, as well as in the dynamic behavior after a brief period of starvation. Further, we were able to delineate the role of individual regulation terms: Three of the known regulatory mechanisms play a major role in the recovery ability of the system. These are (i) the inhibition of PYK by inorganic phosphate, (ii) the activation of PYK by FBP and (iii) the inhibition of PTS by FBP. We showed that in cases of non-recovery the zero-flux metabolic state is an attractor for the system. In the presence of regulation the ability of the system to escape metabolic death is, in the overwhelming number of cases, mirrored by the absence of such attractor.

In summary, sampling unknown kinetic parameters for a kinetic model of a metabolic pathway, such that all sampled parameters give rise to the same metabolic state, can lead to fundamental insights into the control properties of the underlying system. In particular, the emergent control profile exhibits a structure that helps us to draw conclusions about the possible behavior of the system. The probabilistic control profile represents what dynamics we should expect as *typical*, given no further knowledge about specific constraints the system is subject to – a tremendously useful information when interpreting experimentally observed data. In this respect, some of the probabilistic distributions are in line with what we expect given the topology of the network, while other properties are counter-intuitive and therefore point the direction for further investigation. Our approach is particularly suited to investigate the qualitative effects that result from changes in pathway topology, in particular from the presence or absence of regulatory interactions. Our computational approach is straightforward to implement and numerically efficient even for large systems. We therefore expect it to be of high utility also in other studies of metabolic, gene-expression and signal transduction systems.

## Materials and Methods

### From Stoichiometry to Dynamics

Our approach consists of a series of well-defined steps and is based upon related strategies that utilize Monte-Carlo sampling in the study of metabolic networks [22], [24], [26], [28], [30], [48]. The starting point of our analysis is a stoichiometric representation of a metabolic system, as obtained either from textbook knowledge or extracted from genome-scale models of the respective organism. The stoichiometric representation is tested to allow for meaningful flux patterns, for example by an analysis of metabolic flux modes [49] or flux-balance analysis [50]. In addition to stoichiometric dependencies, we assume that the basic regulatory interactions are known. That is, for each enzymatic interconversion there may be a set of metabolites that either inhibits or activates the respective step, albeit with unknown strength. Differing from conventional bottom-up modelling, and following the definitions given in Grimbs et al. [51] and Murabito et al. [28], the subsequent analysis is then based on knowledge of a specific metabolic phenotype of the system. That phenotype is defined by a steady-state flux value for each metabolic reaction, as well as by a set of concentration values for all metabolic intermediates, and has to fulfill two prerequisites. First, its flux distribution must be consistent with the mass-balance constraint. Second, the set of concentration values must be thermodynamically consistent with the directions of the fluxes, namely that given a set of concentration values and the set of equilibrium constants, the Gibbs free energy of all reactions must be negative in the direction indicated by the phenotype's flux distribution. To obtain insights into kinetic properties, each reaction rate is then assigned a rate equation that specifies the dependence of the reaction rate with respect to its substrates and products, as well as with respect to possible allosteric or competitive effectors. In case the actual rate equation of the respective step is unknown, a generic Michaelis-Menten equation is employed [33], [52]. Once these data are assembled, the system of differential equations that determines the dynamic properties of the network has been fully specified except for lacking numerical values for most enzyme-kinetic parameters. As shown previously [28], however, with these definitions and the known metabolic phenotype, it is possible to systematically sample the parametric space, such that the resulting set of enzyme-kinetic parameters is consistent with the known metabolic state. In this way, insight into the typical dynamic properties of a specific metabolic phenotype can be obtained.

### Defining the system and its evaluation

The probabilistic approach used within this work has been described previously [28]. In the following, we briefly outline our workflow. We assume that a metabolic system of interest consisting of 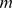 metabolites and 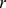 reactions is described by a system of ordinary differential equations of the form, 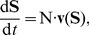
(1)where 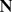 denotes the 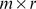 stoichiometric matrix and 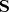 the 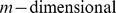 vector of metabolite concentrations. The 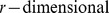 vector 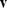 specifies the nonlinear dependencies of the reaction rates as a function of the associated metabolite concentrations and kinetic parameters. To evaluate the dynamics, we assume the existence of a feasible metabolic state, defined by a concentration vector 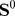 and its associated flux values 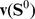, such that 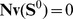. We note that the metabolic state does not necessarily has to be asymptotically stable. The matrices of flux control coefficients 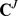 and concentration control coefficients 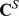 can be expressed as, 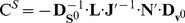
(2)and 
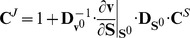
(3)where 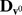 and 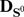 denote diagonal matrices with elements 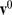 and 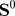 on the diagonal, respectively, 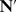 denotes the reduced stoichiometric matrix and 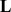 the link matrix. See [28] for details. The Jacobian 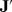 accounts for possible mass conservation and is defined as 
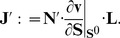
(4)


The control coefficients are only evaluated for stable metabolic states, characterized by an invertible Jacobian with all real parts of the eigenvalues below zero. The information required to evaluate the control coefficients therefore consists of: (i) The stoichiometry of the system, as encoded by 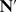 and 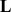; (ii) the metabolic state, as encoded in the matrices 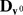 and 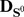; and (iii) the kinetic properties of the reactions, as encoded in the partial derivatives. The partial derivatives are also known as the unscaled elasticity coefficients.

Our probabilistic evaluation of the system is then based on the fact that the metabolic state is often directly experimentally accessible, whereas information about kinetic parameters, and hence the elasticities, is generally lacking. We therefore evaluate the possible values of the unscaled elasticities by drawing random instances of parameters and evaluating the equations for the control coefficients. In particular, we proceed along the following steps: First, each reaction is associated with a kinetic reaction equation. We adopt general Michaelis-Menten kinetics of the form 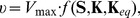
(5)where 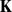 denotes a vector of unknown Michaelis-Menten parameters and 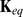 denotes an equilibrium constant. The function 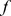 includes terms for possible inhibition and activation. For example, for an unregulated uni-uni reaction 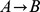, the equation reads 
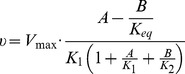
(6)


The number of unknown reaction parameters typically equals the number of associated substrates, products and modifiers (plus 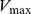 and the equlibrium constant). A full list of rate equations is provided in the Text S2. Second, the kinetic parameters 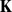 are sampled from intervals chosen according to the associated metabolite concentration, such that 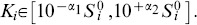
(7)


For each simulation, the set of Michaelis-Menten parameters is chosen at random. All results are reported for 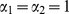, but the results are highly robust for different choices of 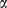 and 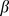. Sampling was linear in log space, i.e., the logarithm of 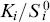 is equidistributed in the interval. Once the parameters are specified, the values of 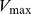 are adjusted so as to deliver the known steady-state flux, 
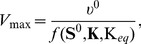
(8)and the derivatives of the reaction equation can be computed. Parameter sampling and the evaluation of control coefficients were repeated 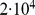 times for each regulatory scenario. The values obtained for the control coefficients were largely robust with respect to different choices of the rate equations. Specifically, the evaluation of the control coefficient only depends on the values attained by the partial derivative, which are primarily determined by the ratio of substrate concentrations with respect to their corresponding Michaelis-Menten constants, as well as by parameter-independent thermodynamic contributions. Using a similar sampling scheme with slightly modified reaction equations is therefore unlikely to result in major deviations with respect to overall control properties [38]. We note that our approach is straightforward to implement and its robustness with respect to different sampling schemes has been tested previously [28]. In particular, the evaluation of the control coefficients does not require explicit kinetic simulation of nonlinear differential equations, making it applicable also for medium- and large-scale systems. The key requirements are knowledge of the network topology, including its regulatory interactions, the metabolic state, as well as the respective thermodynamic equilibrium constants. All code is provided in File S1.

### Fermentation experiments

*L. lactis* cells were grown anaerobically at 37°C in CDM-LAB medium [53]. The medium contained per liter: 1 g K
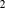
HPO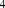, 5 g KH
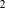
PO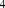, 0.6 g ammonium citrate, 1 g acetate, 0.25 g tyrosine, 0.24 g alanine, 0.125 g arginine, 0.42 g aspartic acid, 0.13 g cysteine, 0.5 g glutamic acid, 0.15 g histidine, 0.21 g isoleucine, 0.475 g leucine, 0.44 g lysine, 0.275 phenylalanine, 0.675 g proline, 0.34 g serine, 0.225 g threonine, 0.05 g tryptophan, 0.325 g valine, 0.175 g glycine, 0.125 g methionine, 0.1 g asparagine, 0.2 g glutamine, 10 g glucose, 0.5 g L-ascorbic acid, 35 mg adenine sulfate, 27 mg guanine, 22 mg uracil, 50 mg cystine, 50 mg xanthine, 2.5 mg D-biotin, 1 mg vitamin B12, 1 mg riboflavin, 5 mg pyridoxamine-HCl, 10 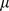
g p-aminobenzoëic acid, 1 mg pantothenate, 5 mg inosine, 1 mg nicotinic acid, 5 mg orotic acid, 2 mg pyridoxine, 1 mg thiamine, 2.5 mg lipoic acid, 5 mg thymidine, 200 mg MgCl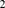, 50 mg CaCl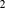, 16 mg MnCl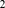, 3 mg FeCl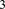, 5 mg FeCl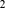, 5 mg ZnSO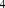, 2.5 mg CoSO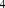, 2.5 mg CuSO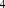, (NH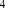)6Mo
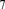
O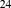. Mid-exponentially grown cells were harvested by centrifugation at 5000 RPM for 10 minutes at room temperature, washed twice with 50 mM MES buffer (pH  =  6.5), and finally suspended in the indicated buffer solution. Anaerobic conditions were established by flushing with nitrogen for 10 min. Glucose (20 mM for *L. lactis*) was added and samples were taken at regular time intervals. 400 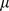
l samples were taken and mixed immediately with 200 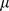
l of a cold perchloric acid (3.5 M) solution. The extracts were kept on ice for maximal 60 minutes. The pH was neutralized with 160 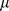
l 2 M KOH. The pH-adjusted samples were centrifuged and the supernatants were stored at −80°C for subsequent analysis. All metabolites were quantified by enzymatic methods coupled to the spectrophotometric determination of NAD(P)H. The strain NZ9000 was used [54].

### Analysis of carbon fluxes

Bacterial dry weight was measured as described previously [55]. External glucose, pyruvate, lactate, formate, acetate, succinate, and ethanol were determined by high-pressure liquid chromatography (HPLC; LKB) with a Rezex organic acid analysis column (Phenomenex) at a temperature of 45°C with 7.2 mM H2SO4 as the eluent, using a RI 1530 refractive index detector (Jasco) and AZUR chromatography software for data integration. Discrimination between d- and l-lactate was performed using a d-/l-lactate assay kit (Megazyme).

### Determination of the metabolic state

Fluxes for lactate, acetate, formate, pyruvate and ethanol were calculated using their fermentation broth concentration, dilution rate (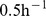) and steady state bacterial cell dry weight. Fluxes are given in Text S1. Steady state intracellular metabolites concentrations were gathered from previously published articles in various journals. The steady state data and its sources are provided in Text S1.

### Determination of the equilibrium constants

The equilibrium constants 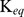 were derived using the following equation, 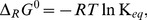
(9)where 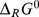 denotes the change in standard Gibbs free energy occurring in the corresponding reaction and RT the gas constant multiplied by the absolute temperature. The value of 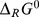 was estimated using, 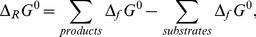
(10)where 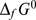 denotes the Gibbs free energy of formation of the different metabolites. The values were adopted from Feist et al. [34], who use the group contribution method developed by Mavrovouniotis [56], [57]. The full list of equilibrium constants is provided in the Text S1. The metabolic state was checked for thermodynamics feasibility. In particular for any reaction 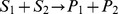 with positive flux, the following relationship must hold at steady state, 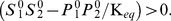
(11)


In Figure 5, we consider the distance 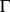 from equilibrium, defined as the ratio 
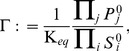
(12)for a reaction that converts a set of substrates 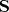 into a set of products 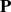. Only considering the positive direction of flux, the value of 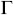 is zero for irreversible reactions and approaches unity for reactions close to equilibrium.

## Supporting Information

Text S1 **A pdf document detailing the experimental procedure and the metabolic state.**
(PDF)

Text S2 **A pdf document providing additional information about computational methods and results.**
(PDF)

File S1 **A zip file providing all code used in the analysis.**
(ZIP)
