# Correction: Monte-Carlo Modeling of the Central Carbon Metabolism of *Lactococcus lactis*: Insights into Metabolic Regulation

**DOI:** 10.1371/journal.pone.0112204

**Published:** 2014-10-23

**Authors:** The PLOS ONE Staff

Figure 3 is a duplicate of Figure 7. The authors have provided the correct Figure 3 here.

**Figure 3:**
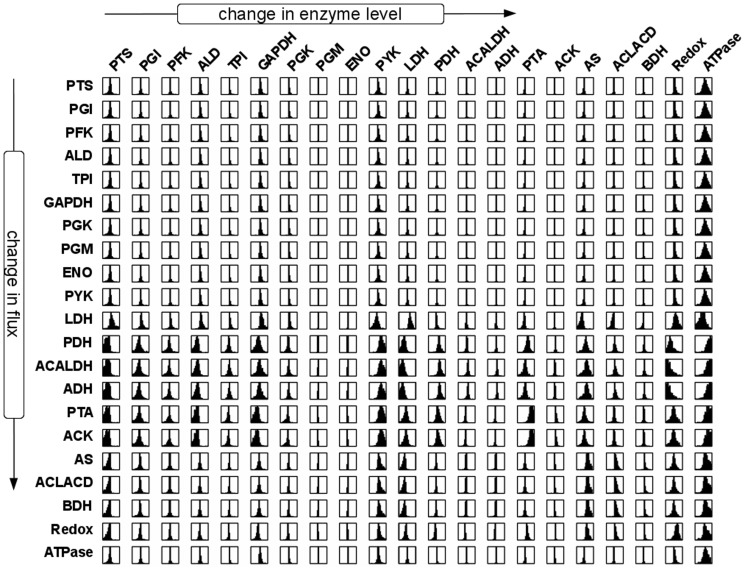
Probabilistic distribution of flux control coefficients. Shown is the distribution of the scaled flux control coefficients corresponding to the pathway model of *L. lactis* central metabolism given in Figure 1. Each plot corresponds to the interval [-1,1] on the abscissa. The diagram in the 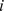
th column and on the 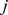
th row gives the distribution of the control coefficient quantifying the extent to which enzyme 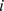 controls the flux through the reaction j. Each distribution provides information about the magnitude and uncertainty of one control coefficient. Narrow distributions indicate control coefficients that do not change appreciably due to parameter sampling, whereas broad distributions indicate that the precise value of the coefficient is more strongly dependent on parameter values. The corresponding sign distribution is shown in Figure 4.
