# Correction: Monte-Carlo Modeling of the Central Carbon Metabolism of *Lactococcus lactis*: Insights into Metabolic Regulation

**DOI:** 10.1371/journal.pone.0116129

**Published:** 2014-12-19

**Authors:** The PLOS ONE Staff

## Notice of Republication

This article was republished on November 19th, 2014, due to Figure 3 being erroneously published as a duplicate of Figure 7. The publisher apologizes for this error. Please download the PDF again to view the corrected article. The originally published, uncorrected article and the republished, corrected article are provided here for reference.

## Supporting Information

File S1 Originally published, uncorrected article
(PDF)

File S2 Republished, corrected article
(PDF)
