# Supplementary material for: Monte-Carlo Modeling of the Central Carbon Metabolism of Lactococcus lactis: Insights into Metabolic Regulation
Source: PLoS One. 2014 Sep 30;9(9):e106453. doi: 10.1371/journal.pone.0106453 (PMC4182131; doi:10.1371/journal.pone.0106453)
Supplement: Text S1 — A pdf document detailing the experimental procedure and the metabolic state. (PDF) [file pone.0106453.s001.pdf]

SUPPLEMENTAL TEXT S1

for

Monte-Carlo Modeling of the Central Carbon Metabolism of Lactococcus Lactis: Insights into Metabolic Regulation

by Murabito et al. (2014)

Determination of extracellular fluxes: Fluxes for lactate, acetate, formate, pyruvate and ethanol were calculated using their fermentation broth concentration, dilution rate (0.5 h^{-1}) and steady state bacterial cell dry weight. Fluxes are shown in Table S1.

Table S1: Extracellular fluxes measured in the current study.

Reaction ID	Chemical equation	Flux (mM/min)	Source
LDH	PYR + NADH → LAC + NAD	4.68E+02	Measured in this study
ADH	ACALD + NADH → ETOH + NAD	5.96E+01	Measured in this study
ACK	ACPH + ADP → ACETATE + ATP	4.67E+01	Measured in this study
ACLACD	ACLAC → Acetoin	1.42E+02	Measured in this study
BDH	Acetoin + NADH → Butanediol + NAD	1.42E+02	Measured in this study
PTS	GLCo + PEP → G6P + PYR	4.29E+02	Measured in this study

Intracellular metabolites: Steady state intracellular metabolites concentrations were gathered from previously published articles in various journals. The steady state data and it sources are given in Table S2.

Table S2: Steady state concentrations of intracellular metabolites.

Metabolite	Conc. (mM)	Source
PYR	9.00	Ana et al., (2000)
PHI	5.00	Current study
NAD	8.40	Garrigues et al., (1997)
ADP	6.10	Current study
G6P	2.50	Garrigues et al., (1997)
GAP	2.40	Ana et al., (2000)
COA	0.08	
PEP	2.10	Garrigues et al., (1997)
DHAP	6.12	Ana et al., (2000)
Acetoin	0.1	
G3P	0.6	Garrigues et al., (1997)
F6P	5.0	Ana et al., (2000)
ACALD	0.0004	
ACPH	0.00	
G2P	0.5	Garrigues et al., (1997)
G13P2	0.6	Garrigues et al., (1997)
ACLAC	0.1	
FBP	45.0	Ana et al., (2000)
NADH	0.7	Garrigues et al., (1997)
ATP	3.9	Current study
ACCOA	0.92	Lall et al., (2011)
LAC	75	Ana et al., (2002)
FMT	0.1	Ana et al., (2002)
ETOH	0.1	Ana et al., (2002)
Butanediol	0.1	Current study
ACETATE	0.1	Ana et al., (2002)
GLCo	29	Current study
GLC	0.1	Model calculated
BIOM	1.0	Assumed
PHIo	7.0	

Metabolic regulations: Regulation of various glycolytic enzymes by intra/extra cellular metabolites was adopted from previously published reports in literature. Details of regulations are shown in Table S3 with parameters and it source.

Table S3: List of metabolic regulations in the glycolysis of *L. lactis*:

Reaction	Regulator	Regulation	Source
re21: GLCo + PEP = G6P + PYR	FBP	Inhibitor	Neves et al., (2005); Voit et al., (2006)
re7: GAP + NAD + PHI = G13P2 + NADH	NADH	Inhibitor	Neves et al., (2005); Voit et al., (2006)
re11: PRP + ADP = PYR + ATP	PHI (Pi)	Inhibitor	Neves et al., (2005)
	FBP	Activator	Neves et al., (2005); Voit et al., (2006)
re13: PYR + COA = ACCOA + FMT	DHAP	Inhibitor	Neves et al., (2005)
	GAP	Inhibitor	Neves et al., (2005); Voit et al., (2006)
re15: ACALD + NADH = ETOH + NAD	ATP	Inhibitor	Neves et al., (2005)
re12: PYR + NADH = LAC + NAD	PHI	Inhibitor	Neves et al., (2005)
	FBP	Activator	Neves et al., (2005)
	NADH/NAD ratio	Inhibitor	Garrigues et al., (1997)

Table S4 – List of equilibrium constants. The equilibrium constants are calculated using group contribution method.

Reaction ID	Chemical equation	Keq
GLT	GLCo → GLC	1.00E+00
HXK	GLC + ATP → G6P + ADP	5.81E+02
PGI	G6P → F6P	3.71E+00
PFK	F6P + ATP → FBP + ADP	5.81E+02
ALD	FBP → DHAP + GAP	1.97E+00
TPI	DHAP → GAP	1.00E+00
GAPDH	GAP + PHI + NAD → G13P2 + NADH	1.67E+00
PGK	G13P2 + ADP → G3P + ATP	1.53E+02
PGM	G3P → G2P	9.98E-01
ENO	G2P → PEP	4.59E+00
PYK	PEP + ADP → PYR + ATP	4.83E+06
LDH	PYR + NADH → LAC + NAD	3.19E+04
PDH	PYR + CoA → ACCoA + FMT	5.47E+03
ACALDH	ACCoA + NADH → ACALD + NAD + CoA	3.49E-04
ADH	ACALD + NADH → ETOH + NAD	1.51E+04
PTA	ACCoA + PHI → ACPH + CoA	1.52E-03
ACK	ACPH + ADP → ACETATE + ATP	4.79E+03
ATPase	ATP → ADP + PHI	9.99E+04
AS	PYR → ACLAC	4.11E+07
ACLACD	ACLAC → Acetoin	3.19E+01
BDH	Acetoin + NADH → Butanediol + NAD	4.98E+02
Redox Balance	NAD → NADH	1.00E+05
PTS	GLCo + PEP → G6P + PYR	4.83E+06

References:

Ana R. Neves, Ana Ramos, Claire Shearman, Michael J. Gasson, Jonas S. Almeida and Helena Santos (2000). Metabolic characterization of Lactococcus lactis deficient in lactate dehydrogenase using in vivo 13C-NMR. Eur. J. Biochem. 267, 3859-3868.

Ana Rute Neves, Rita Ventura, Nahla Mansour, Claire Shearman, Michael J. Gasson, Christopher Maycock, Ana Ramos, and Helena Santos (2002). Is the Glycolytic Flux in Lactococcus lactis Primarily Controlled by the Redox Charge? KINETICS OF NAD AND NADH POOLS DETERMINED IN VIVO BY 13C NMR. J Biol Chem 277(31), 28088–28098.

Garrigues C, Loubiere P, Lindley ND, and Cocaign-Bousquet M. Control of the Shift from Homolactic Acid to Mixed-Acid Fermentation in Lactococcus lactis: Predominant Role of the NADH/NAD Ratio (1997). J Bacteriol. 179 (17): 5282–5287.

Mavrovouniotis, M. L. (1990). Group contributions for estimating standard Gibbs energies of formation of biochemical-compounds in aqueous-solution. Biotechnol. Bioeng. 36:1070–1082.

Mavrovouniotis, M. L. 1991. Estimation of standard Gibbs energy changes of biotransformations. J. Biol. Chem. 266:14440–14445.

Neves AR, Pool WA, Kok J, Kuipers OP, Santos H (2005). Overview on sugar metabolism and its control in *Lactococcus lactis* - the input from in vivo NMR. FEMS Microbiol Rev 29(3): 531-554.

Voit EO, Almeida J, Marino S, Lall R, Goel G, Neves AR and Santos H (2006). Regulation of glycolysis in Lactococcus lactis: an unfinished systems biological case study. IEE Proc.-Syst. Biol. 153(4); 286-297.

Feist AM, Henry CS, Reed JL, Krummenacker M, Joyce AR, Karp PD, Broadbelt LJ, Hatzimanikatis V & Palsson B Ø (2007). A genome-scale metabolic reconstruction for *Escherichia coli* K-12 MG1655 that accounts for 1260 ORFs and thermodynamic information. *Molecular Systems Biology* **3:121**

Lall R., Donohue T. J. and Mitchell J.C. (2011). Optimizing ethanol production selectivity. Mathematical and Computer Modelling 53(7-8): 1363-1373.
