# Supplementary material for: Monte-Carlo Modeling of the Central Carbon Metabolism of Lactococcus lactis: Insights into Metabolic Regulation
Source: PLoS One. 2014 Sep 30;9(9):e106453. doi: 10.1371/journal.pone.0106453 (PMC4182131; doi:10.1371/journal.pone.0106453)
Supplement: Text S2 — A pdf document providing additional information about computational methods and results. (PDF) [file pone.0106453.s002.pdf]

Supplemental Text S2

for

Monte-Carlo Modeling of the Central Carbon Metabolism of *Lactococcus lactis*: Insights into Metabolic Regulation

by Murabito et al. (2014)

The Kinetic Rate Equations

In the following, we provide a comprehensive list of rate equations used within our analysis. We note that kinetic parameters with identical name across different kinetic equations (such as V_{max} or K_{eq}) are meant to represent different parameters. In total, the system of differential equations encompasse 77 unknown Michaelis-Menten constants. We note that our choice of rate equations takes inhibition between substrates and products into account.

$$v_{PTS} = \left(\frac{K_i^{FBP}}{K_i^{FBP} + FBP}\right) \cdot \frac{V_{max} \cdot \frac{GLCo \cdot PEP}{k_M^{GLCo} \cdot k_M^{PEP}} \cdot \left(1 - \frac{G6P \cdot PYR}{GLCo \cdot PEP \cdot K_{eq}}\right)}{\left(1 + \frac{GLCo}{k_M^{GLCo}}\right) \cdot \left(1 + \frac{PEP}{k_M^{PEP}}\right) + \left(1 + \frac{G6P}{k_M^{G6P}}\right) \cdot \left(1 + \frac{PYR}{k_M^{PYR}}\right) - 1}$$

$$v_{PGI} = \frac{V_{max} \cdot \left(\frac{G6P}{k_M^{G6P}}\right) \cdot \left(1 - \frac{F6P}{G6P \cdot K_{eq}}\right)}{\left(1 + \frac{G6P}{k_M^{G6P}} + \frac{F6P}{k_M^{F6P}}\right)}$$

$$v_{PFK} = \frac{V_{max} \cdot \frac{F6P \cdot ATP}{k_M^{F6P} \cdot k_M^{ATP}} \cdot \left(1 - \frac{FBP \cdot ADP}{F6P \cdot ATP \cdot K_{eq}}\right)}{\left(1 + \frac{F6P}{k_M^{F6P}} + \frac{FBP}{k_M^{FBP}}\right) \cdot \left(1 + \frac{ATP}{k_M^{ATP}} + \frac{ADP}{k_M^{ADP}}\right)}$$

$$v_{ALD} = \frac{V_{max} \cdot \frac{FBP}{k_M^{FBP}} \cdot \left(1 - \frac{DHAP \cdot GAP}{FBP \cdot K_{eq}}\right)}{\left(1 + \frac{FBP}{k_M^{FBP}}\right) + \left(1 + \frac{DHAP}{k_M^{DHAP}}\right) \cdot \left(1 + \frac{GAP}{k_M^{GAP}}\right) - 1}$$

$$v_{TPI} = \frac{V_{max} \cdot \frac{DHAP}{k_M^{DHAP}} \cdot \left(1 - \frac{GAP}{DHAP \cdot K_{eq}}\right)}{1 + \frac{GAP}{k_M^{GAP}} + \frac{DHAP}{k_M^{DHAP}}}$$

$$v_{GAPDH} = \left(\frac{K_i^{NADH}}{K_i^{NADH} + NADH}\right) \cdot \frac{V_{max} \cdot \frac{GAP \cdot NAD \cdot Pi}{k_M^{GAP} \cdot k_M^{NAD} \cdot k_M^{Pi}} \cdot \left(1 - \frac{G13P2 \cdot NADH}{GAP \cdot NAD \cdot Pi \cdot K_{eq}}\right)}{\left(1 + \frac{NAD}{k_M^{NAD}} + \frac{NADH}{k_M^{NADH}}\right) \cdot \left(1 + \frac{GAP}{k_M^{GAP}} + \frac{G13P2}{k_M^{G13P2}} + \frac{Pi}{k_M^{Pi}}\right)}$$

$$v_{PGK} = \frac{V_{max} \cdot \frac{G13P2 \cdot ADP}{k_M^{G13P2} \cdot k_M^{ADP}} \cdot \left(1 - \frac{G3P \cdot ATP}{G13P2 \cdot ADP \cdot K_{eq}}\right)}{\left(1 + \frac{G13P2}{k_M^{G13P2}} + \frac{G3P}{k_M^{G3P}}\right) \cdot \left(1 + \frac{ADP}{k_M^{ADP}} + \frac{ATP}{k_M^{ATP}}\right)}$$

$$v_{PGM} = \frac{V_{max} \cdot \frac{G3P}{k_M^{G3P}} \cdot \left(1 - \frac{G2P}{G3P \cdot K_{eq}}\right)}{1 + \frac{G3P}{k_M^{G3P}} + \frac{G2P}{k_M^{G2P}}}$$

$$v_{ENO} = \frac{V_{max} \cdot \frac{G2P}{k_M^{G2P}} \cdot \left(1 - \frac{PEP}{G2P \cdot K_{eq}}\right)}{1 + \frac{G2P}{k_M^{G2P}} + \frac{PEP}{k_M^{PEP}}}$$

$$v_{PYK} = \left(\frac{K_i^{Pi}}{K_i^{Pi} + Pi}\right) \cdot \left(\frac{FBP}{K_a^{FBP} + FBP}\right) \cdot \frac{V_{max} \cdot \frac{PEP \cdot ADP}{k_M^{PEP} \cdot k_M^{ADP}} \cdot \left(1 - \frac{PYR \cdot ATP}{PEP \cdot ADP \cdot K_{eq}}\right)}{\left(1 + \frac{PEP}{k_M^{PEP}} + \frac{PYR}{k_M^{PYR}}\right) \cdot \left(1 + \frac{ADP}{k_M^{ADP}} + \frac{ATP}{k_M^{ATP}}\right)}$$

$$v_{LDH} = \left(\frac{K_i^{NADH/NAD}}{K_i^{NADH/NAD} + \frac{NADH}{NAD}}\right) \cdot \left(\frac{FBP}{K_a^{FBP} + FBP}\right) \cdot \left(\frac{K_i^{Pi}}{K_i^{Pi} + Pi}\right) \cdot \frac{V_{max} \cdot \frac{PYR \cdot NADH}{k_M^{PYR} \cdot k_M^{NADH}} \cdot \left(1 - \frac{LAC \cdot NAD}{PYR \cdot NADH \cdot K_{eq}}\right)}{\left(1 + \frac{PYR}{k_M^{PYR}} + \frac{LAC}{k_M^{LAC}}\right) \cdot \left(1 + \frac{NADH}{k_M^{NADH}} + \frac{NAD}{k_M^{NAD}}\right)}$$

$$v_{PDH} = \left(\frac{K_i^{GAP}}{K_i^{GAP} + GAP}\right) \cdot \left(\frac{K_i^{DHAP}}{K_i^{DHAP} + DHAP}\right) \cdot \frac{V_{max} \cdot \frac{PYR \cdot COA}{k_M^{PYR} \cdot k_M^{COA}} \cdot \left(1 - \frac{ACCOA \cdot FMT}{PYR \cdot COA \cdot K_{eq}}\right)}{\left(1 + \frac{PYR}{k_M^{PYR}} + \frac{COA}{k_M^{COA}}\right) \cdot \left(1 + \frac{ACCOA}{k_M^{ACCOA}} + \frac{FMT}{k_M^{FMT}}\right)}$$

$$v_{ACALDH} = \frac{V_{max} \cdot \frac{ACCOA \cdot NADH}{k_M^{ACCOA} \cdot k_M^{NADH}} \cdot \left(1 - \frac{ACALD \cdot NAD \cdot COA}{ACCOA \cdot NADH \cdot K_{eq}}\right)}{\left(1 + \frac{ACCOA}{k_M^{ACCOA}} + \frac{NADH}{k_M^{NADH}}\right) \cdot \left(1 + \frac{ACALD}{k_M^{ACALD}} + \frac{NAD}{k_M^{NAD}} + \frac{COA}{k_M^{COA}}\right)}$$

$$v_{ADH} = \left(\frac{K_i^{ATP}}{ATP + K_i^{ATP}}\right) \cdot \frac{V_{max} \cdot \frac{ACALD \cdot NADH}{k_M^{ACALD} \cdot k_M^{NADH}} \cdot \left(1 - \frac{EtOH \cdot NAD}{ACALD \cdot NADH \cdot K_{eq}}\right)}{\left(1 + \frac{ACALD}{k_M^{ACALD}} + \frac{EtOH}{k_M^{EtOH}}\right) \cdot \left(1 + \frac{NADH}{k_M^{NADH}} + \frac{NAD}{k_M^{NAD}}\right)}$$

$$v_{PTA} = \frac{V_{max} \cdot \frac{ACCOA \cdot Pi}{k_M^{ACCOA} \cdot k_M^{Pi}} * \left(1 - \frac{ACPH \cdot COA}{ACCOA \cdot Pi \cdot K_{eq}}\right)}{\left(1 + \frac{ACCOA}{k_M^{ACCOA}}\right) \cdot \left(1 + \frac{Pi}{k_M^{Pi}}\right) + \left(1 + \frac{COA}{k_M^{COA}}\right) \cdot \left(1 + \frac{ACPH}{k_M^{ACPH}}\right) - 1}$$

$$v_{ACK} = \frac{V_{max} \cdot \frac{ACPH \cdot ADP}{k_M^{ACPH} \cdot k_M^{ADP}} \cdot \left(1 - \frac{ACETATE \cdot ATP}{ACPH \cdot ADP \cdot K_{eq}}\right)}{\left(1 + \frac{ACPH}{k_M^{ACPH}} + \frac{ACETATE}{k_M^{ACETATE}}\right) \cdot \left(1 + \frac{ADP}{k_M^{ADP}} + \frac{ATP}{k_M^{ATP}}\right)}$$

$$v_{ALS} = \frac{V_{max} \cdot \frac{PYR}{k_M^{PYR}} \cdot \left(1 - \frac{ACLAC}{PYR \cdot K_{eq}}\right)}{1 + \frac{PYR}{k_M^{PYR}} + \frac{ACLAC}{k_M^{ACLAC}}}$$

$$v_{ACALDH} = \frac{V_{max} \cdot \frac{ACLAC}{k_M^{ACLAC}} \cdot \left(1 - \frac{Acetoin}{ACLAC \cdot K_{eq}}\right)}{1 + \frac{Acetoin}{k_M^{Acetoin}} + \frac{ACLAC}{k_M^{ACLAC}}}$$

$$v_{BDH} = \frac{V_{max} \cdot \frac{Acetoin \cdot NADH}{k_M^{Acetoin} \cdot k_M^{NADH}} \cdot \left(1 - \frac{Butanediol \cdot NAD}{Acetoin \cdot NADH \cdot K_{eq}}\right)}{\left(1 + \frac{Acetoin}{k_M^{Acetoin}} + \frac{Butanediol}{k_M^{Butanediol}}\right) \cdot \left(1 + \frac{NAD}{k_M NAD} + NADH k_M^{NADH}\right)}$$

$$v_{RedoxBalance} = \frac{V_{max} \cdot \frac{NADH}{k_M^{NADH}} \cdot \left(1 - \frac{NAD}{NADH \cdot K_{eq}}\right)}{1 + \frac{NADH}{k_M^{NADH}} + \frac{NAD}{k_M^{NAD}}}$$

$$v_{ATPase} = \frac{V_{max} \cdot \frac{ATP}{k_M^{ATP}}}{1 + \frac{ATP}{k_M^{ATP}}}$$

Selected Time Courses of Metabolite Concentrations

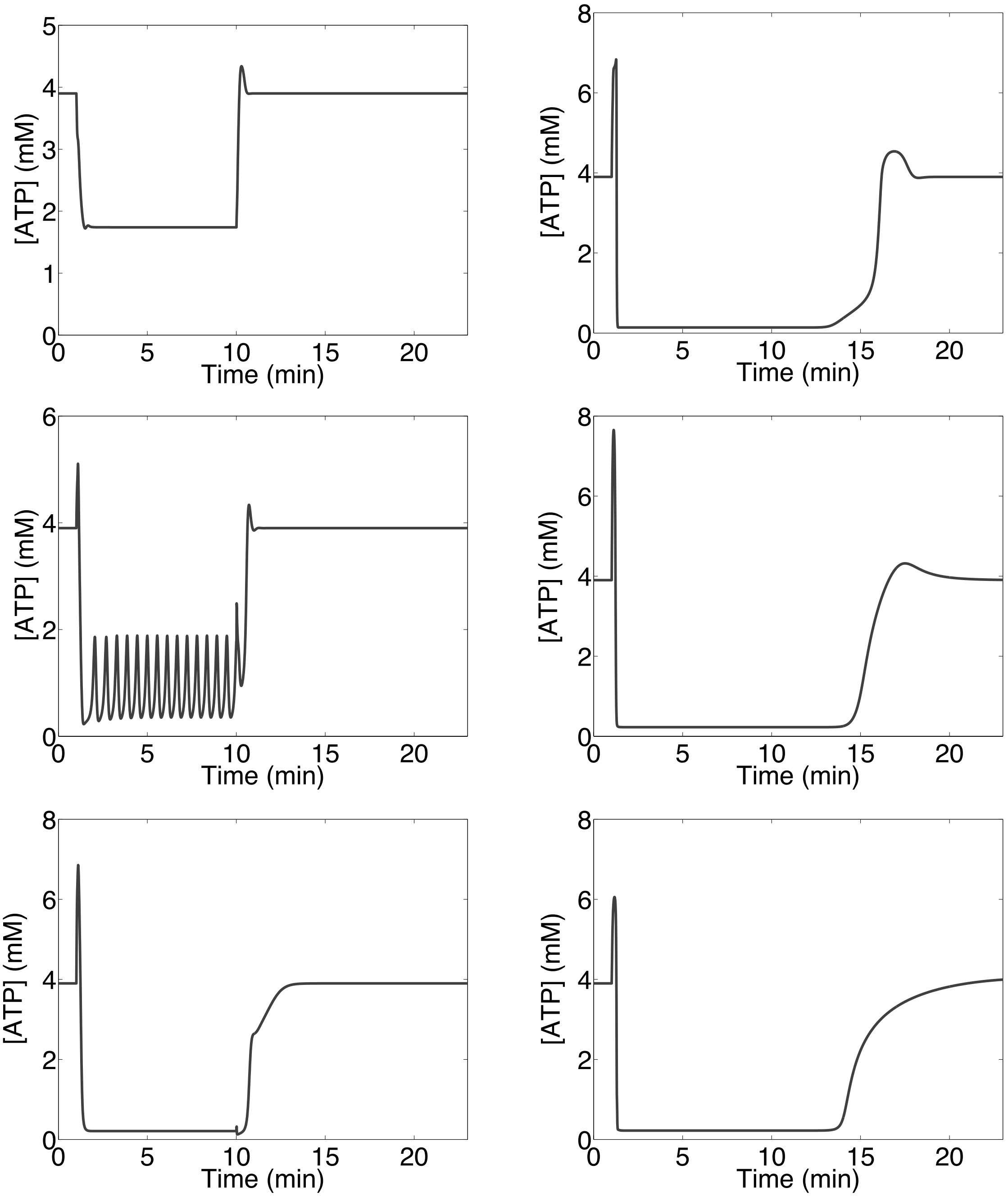

Figure S1: Examples of time courses for regulated (left column) and non-regulated (right column) systems.

Fig. S1 shows different behaviours of the systems depending on the specific set of sampled parameter values and the presence or lack of regulation. The ATP concentration during starvation can assume a wider variety of values in the presence of regulation and can also show oscillations. The average value of ATP concentration during starvation is 0.53mM for regulated systems versus 0.11mM for non-regulated systems. A common feature for both regulated and non-regulated system is the presence of a peak in ATP concentration at the time in which the starvation start (minute 1.0). The presence of this peak can be explained by the fact that, once external glucose has been deprived, the first part of glycolysis slows down for lack of its substrate resulting in a slower consumption of ATP by PFK. The downstream part of glycolysis might have a delay in responding to this stress, hence there will be a time-span during which ATP is produced faster than it is consumed.

Bistability and Hysteresis

As detailed in the main text, both in presence and lack of regulation we registered cases in which the system was characterised by bistability. For example, after restoring the pre-starvation level of external glucose, the 0.7% of the regulated systems and the 1.2% of the unregulated systems reached a new steady-state concentration of APT which was: (a) different from the original value within the 10% of tollerance and (b) sensibly different from zero (> 0.5mM). This phenomenon was in most cases mirrored by the hysteresys curves of such bistable systems. However, we also detected cases in which there was a discrepancy between the time course and what the hysteresys curve seemed to suggest. Fig. S3 shows two of such occurrences for the regulated systems.

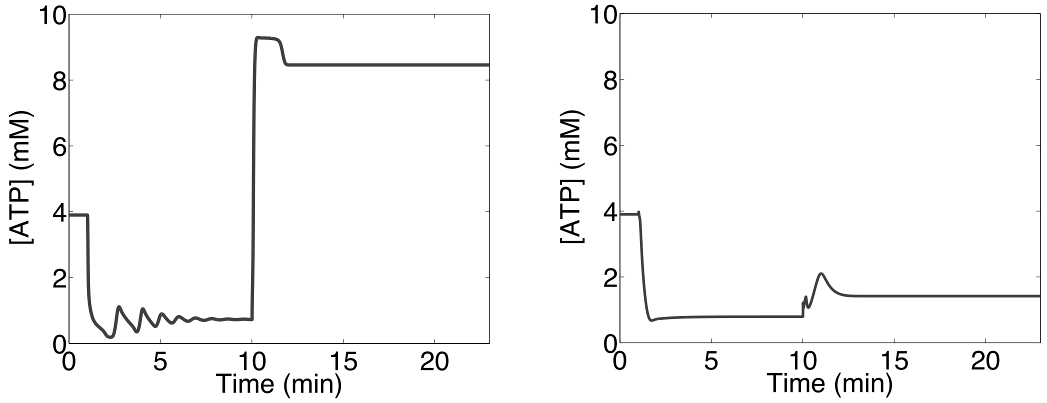

Figure S2: Bistability of some of the regulated systems. The ATP concentration at steady-state after the recovering time is different from the pre-starvation value (within the 10% of tolerance) and from zero (> 0.5mM).

We explored this phenomenon further by generating hysteresis curves where we gradually changed the concentration of external glucose as external parameter.

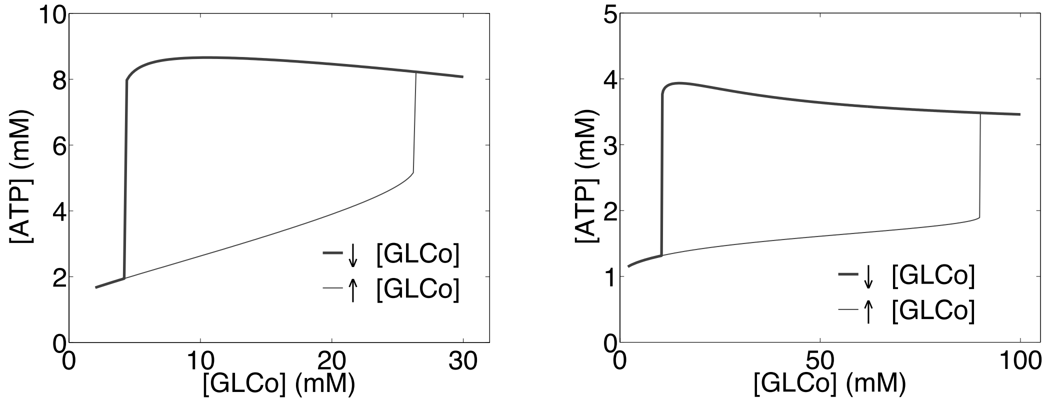

Figure S3: Hysteresis curves of the models referred to in Fig. S1. The ATP concentration at steady-state was recorded while scanning [GLCo] as external parameter.

Fig. S3 shows such hysteresis curves for the same models referred to in Fig. S2. In the case shown in Fig. S2-a and Fig. S3-a, we note that before starvation the concentration of ATP is on the lower side of the hysteresis curve, where the external glucose concentration of 20 mM corresponds to an ATP concentration of about 4 mM. After starvation the ATP concentration increases very steeply, reaching a peak of over 9 mM. From there the system relaxes to the new steady-state with the concentration of ATP decreasing until it reaches the upper part of the hysteresis curve, where the restored concentration of external glucose (20 mM) corresponds to a higher level of ATP (= 8.2 mM).

A Second Metabolic State

In addition to the results presented within the main text, we evaluated the control properties of a second metabolic state, with concentrations and fluxes given in Table S4. The second state was obtained using an explicit kintic model where all Michaelis-Menten parameters are set to values that are identical to the respective concentrations. Using this model, the value of external glucose was lowered to 2mM and the resulting steady state was recorded. In contrast to the metabolic state evalauted within the main text, the second state is therefor not directly based on experimentally acquired data. Nonetheless, analysis of the state allows for some conclusions with respect to typical control properties.

Reaction step	Flux (mM/min)	Species	Conc (mM)
PTS	3.21E+02	PYR	1.12E+01
PGI	3.21E+02	NAD	8.13E+00
PFK	3.21E+02	ADP	7.29 E+00
ALD	3.21E+02	GAP	1.44E+00
TPI	3.21E+02	COA	8.29E-02
GAPDH	6.41E+02	PEP	3.52E+01
PGK	6.41E+02	DHAP	3.45E+00
PGM	6.41E+02	Pi	2.10E+01
ENO	6.41E+02	Acetoin	8.53E-02
PYK	3.21E+02	G3P	8.82E+00
LDH	1.25E+02	F6P	3.75E+00
PDH	2.00E+02	ACALD	1.00E-04
ACALDH	9.92E+01	G6P	1.48E+01
ADH	9.92E+01	G2P	8.02E+00
PTA	1.00E+02	ACPH	2.80E-05
ACK	1.00E+02	ACLAC	1.09E-01
ALS	1.58E+02	FBP	1.89E+01
ACLACD	1.58E+02	G13P2	2.38E+00
BDH	1.58E+02	ATP	2.71E+00
Redox Balan.	1.60E+02	ACCOA	9.17E-01
ATPase	7.41E+02	NADH	9.72E-01
		LAC	7.500E+01
		FMT	1.000E-01
		EtOH	1.000E-01
		Butanediol	1.000E-01
		ACETATE	1.000E-01
		GLCo	2.000E+00

Table S4: List of fluxes and metabolite concentrations defining a second metabolic state. This metabolic phenotype was used to show how a different choice for the state of reference results in different results, mainly with respect to some of the flux control distributions. The results obtained using this second metabolic state are not shown in the main text and are presented in the following section of Supplemental material.

Control properties of the Second Metabolic State

The probabilistic control analysis of the regulated and unregulated system was performed on the second metabolic state characterised by a low concentration of external glucose (see previous section for the complete list of concentrations and fluxes). For this second metabolic state we again observed that the presence of regulation was associated with an increased stability. In particular almost 98% of the regulated systems resulted to be stable versus the 84% of the non-regulted case.

We also notice that the control of the PTS on the glycolytic flux is positive in the second metabolic state for the regulated case . Fig. S5 shows the probabilistic sign distribution of flux control coefficients for regulated and non-regulated systems in the second metabolic state.

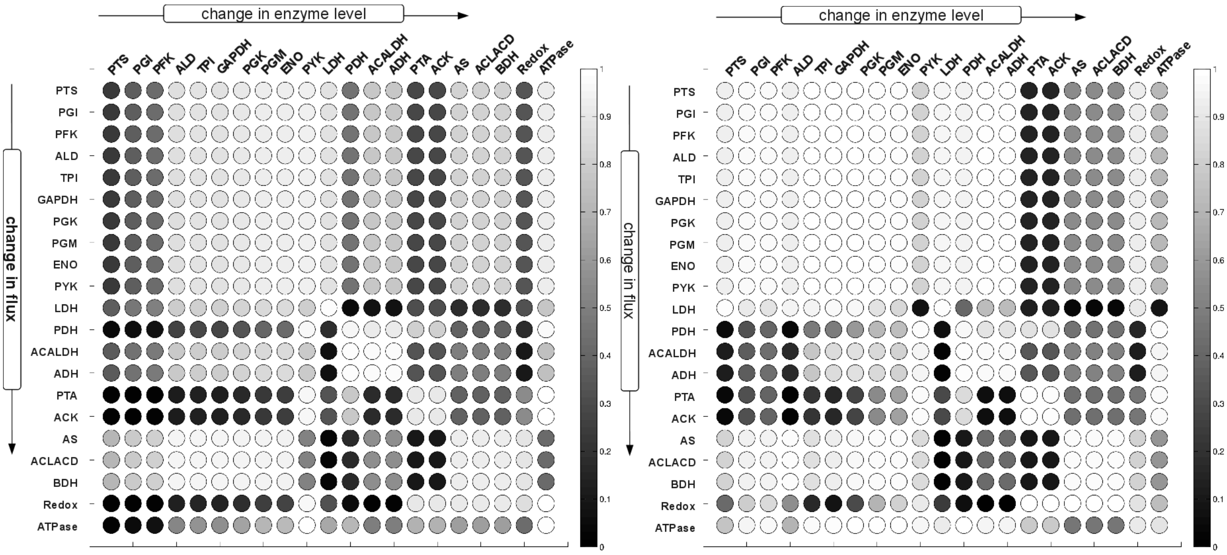

Figure S5: Probabilistic sign distribution of the flux control coefficients for non-regulated (left panel) and regulated (right panel) systems in the second metabolic state. As in Fig.4 of the main text, the shade of each entry represents the percentage of the corresponding control coefficients that are positive.

Figures S6A and S6B show the probabilistic distributions of the flux control coefficients for regulated and non-regulated systems in the second metabolic state. In this case the differences between presence and absence of regulation are more pronounces than in the metabolic state referred to in the main text. In particular in the presence of regulation the distributions tend to be much slimmer, showing how the regulatory mechanisms substantially contribute in confining the value of the control properties of the system. This suggest that the action of the regulation is even more pronounced in a state with low glucose.

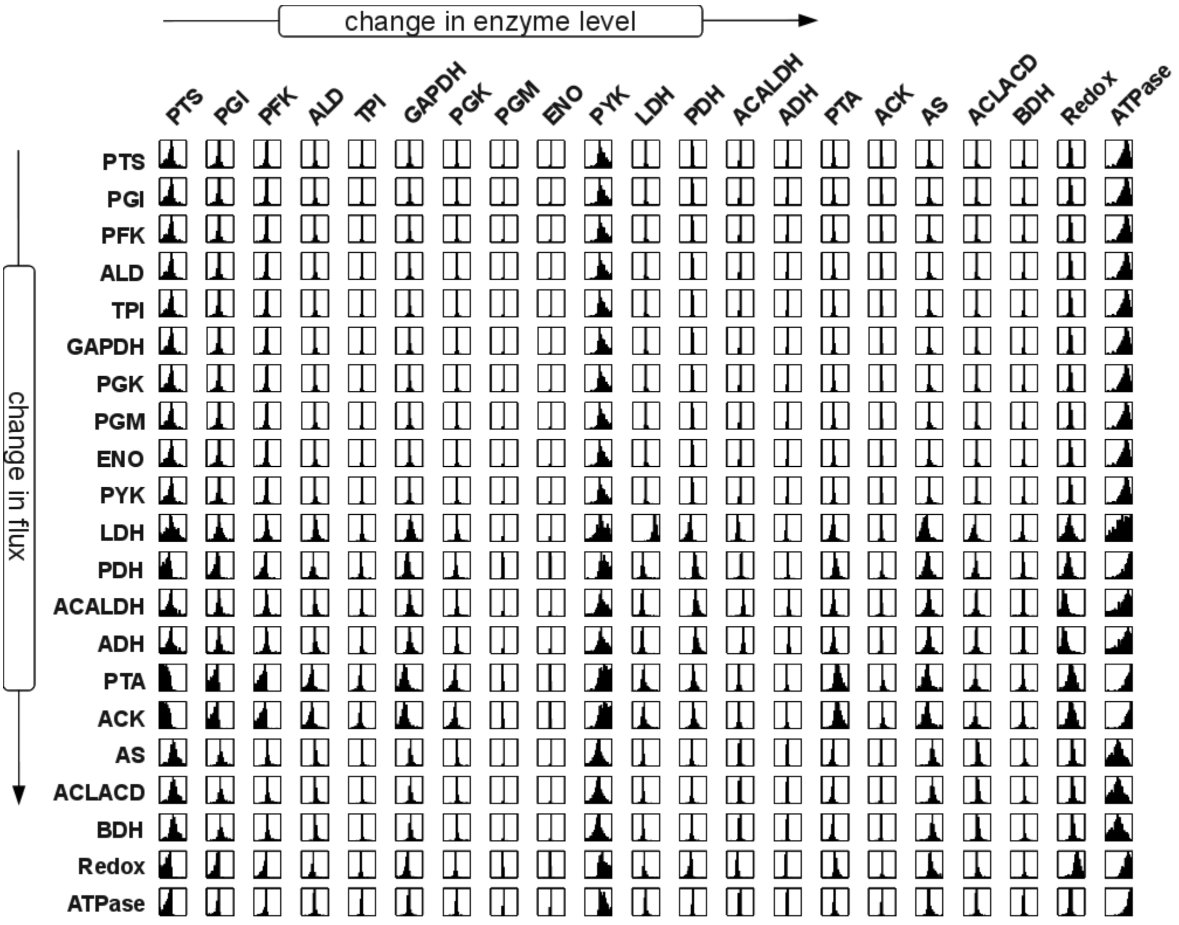

Figure S6A: Probabilistic distribution of the flux control coefficients for the non-regulated system in the second metabolic state. As in Fig.3 of the main text, the distributions refer to the scaled flux control coefficients corresponding to the pathway map of L. lactis central metabolism. Columns denote changes in enzyme levels and rows the respective relative change in flux.

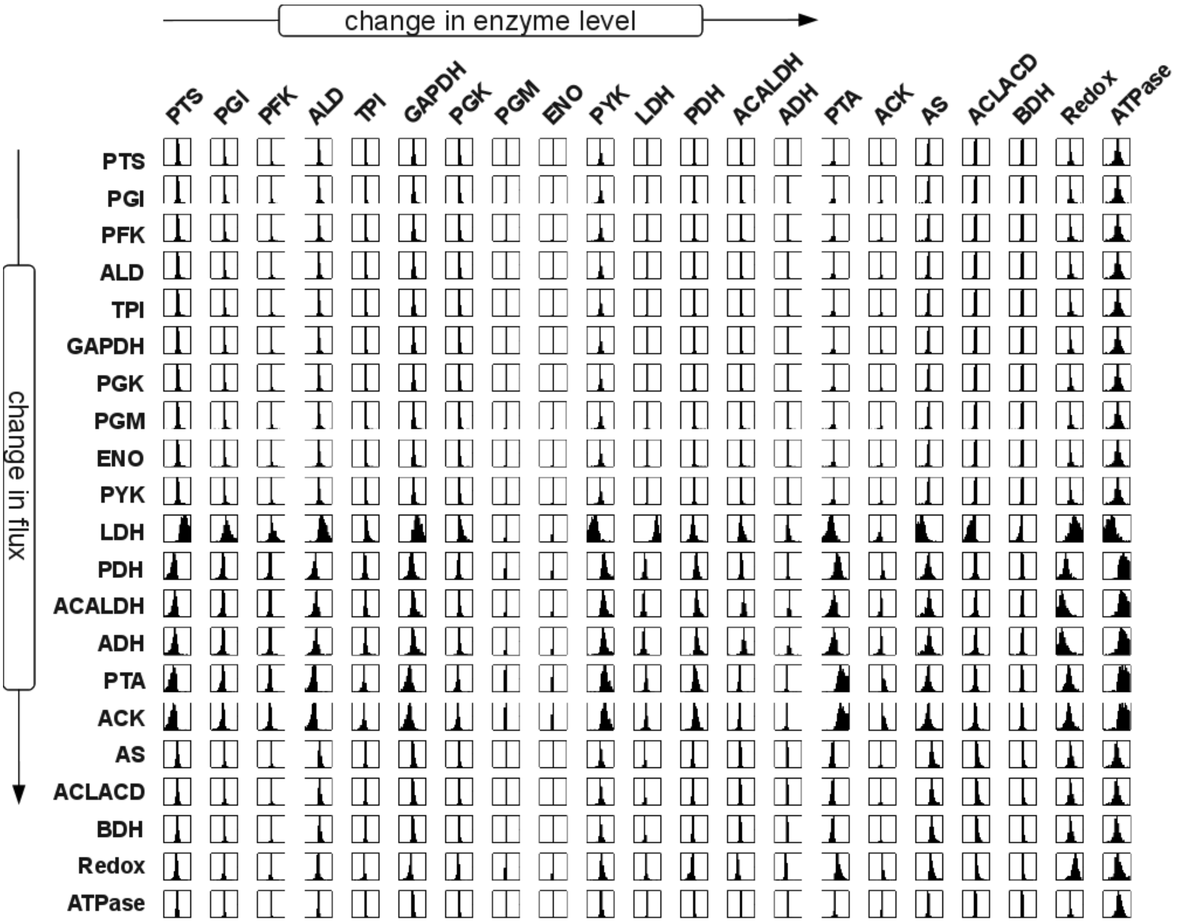

Figure S6B: Probabilistic distribution of the flux control coefficients for the regulated system in the second metabolic state. As in Fig.3 of the main text, the distributions refer to the scaled flux control coefficients corresponding to the pathway map of L. lactis central metabolism. Columns denote changes in enzyme levels and rows the respective relative change in flux.

Convergence of Sampled Control Coefficients

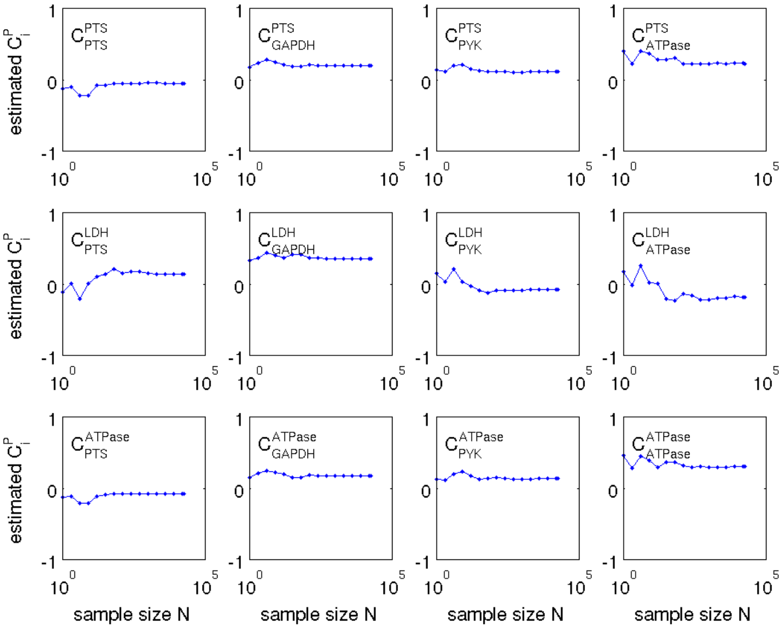

Figure S7A: Convergence of selected control coefficients as a function of sample size. Shown is the median of 12 selected flux control coefficient C_i^P as a function of the number N of samples used for its estimation, with $N = 1, \ldots, 2 \cdot 10^4$. We note that the scale on the x-axis is logarithmic. For sample sizes larger than a few hundred samples, no appreciable variation is observed. The figure corresponds to the control coeeficients shown in Figure 3 of the main text (full system, including regulation).

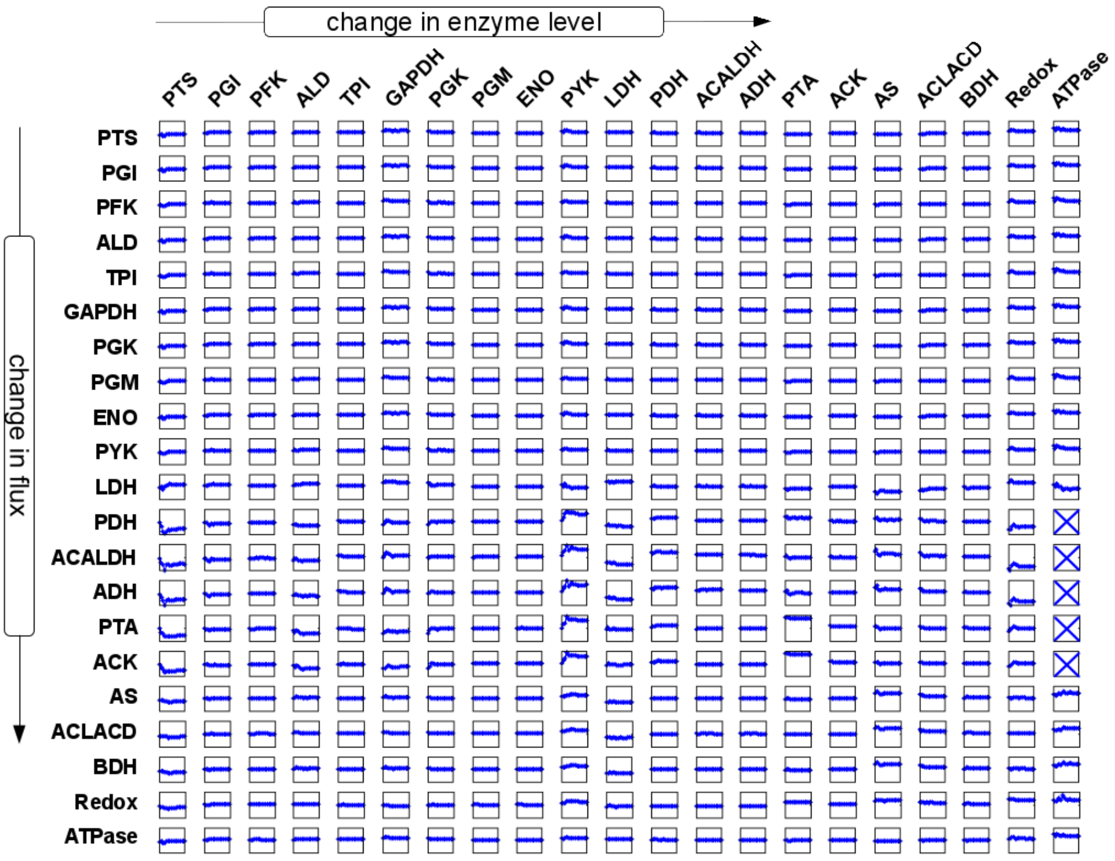

Figure S7B: Convergence of control coefficients as a function of sample size. The figure corresponds to the flux control coeeficients shown in Figure 3 of the main text (full system, including regulation). Shown is the convergence of the median of all control coefficients as a function of sample size. The scaling of the axes is identical to those of Figure S7A. All axes span the interval $[-1, 1]$ on the y-axis and $N = 1, \ldots, 2 \cdot 10^4$ on the x-axis. The latter is scaled logarithmically (See Figure S7A for labels). The flux control coefficient of changing the ATPase on five fluxes (PDH, ACALDH, ADH, PTA, ACK) is larger than one. These values are not shown, convergence properties are similar to those shown.

Recovery in the absence of regulation

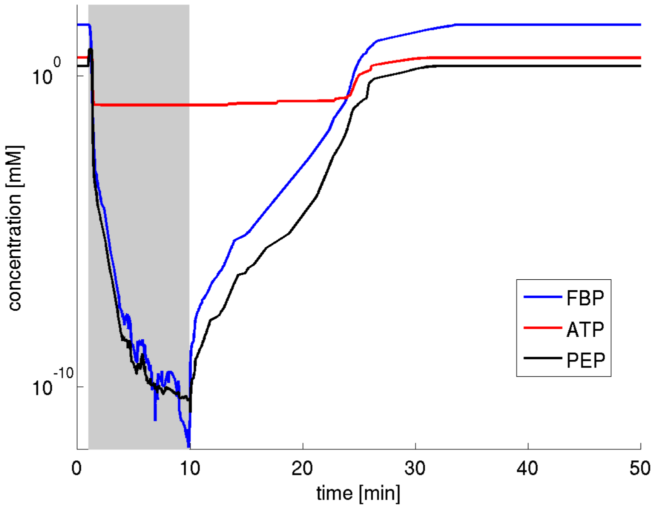

Figure S8: Time-courses of intermediate metabolites. The median of the concentration of FBP, ATP, and PEP following a withdrawal of external glucose at $t = 1$min in models that do not posses regulatory interactions but are nonetheless able to recover after a period of starvation. The figure corresponds to Figure 11 of the main text. In contrast to Figure 11, all intermediate concentrations, including PEP, drop to low values after withdrawal of glucose. With the restoration of external glucose at $t = 10$min, PEP and FBP slowly rise while it takes until $t \approx 25$ min before the concentration of ATP is restored to pre-starvation levels. The figure clearly shows that while fine-tuning of parameters might likewise, at least for a small subset of parameters, result in recovery after starvation, mechanism is very different from the recovery induced by feedforward activation.

Abbreviations and Notation

Lactic acid bacteria, LAB; Generally Regarded As Safe, GRAS; Adenosine-triphosphate, ATP; Adenosine-diphosphate, ADP; free inorganic phosphate, Pi; Nicotinamide adenine dinucleotide, NAD; Reduced nicotinamide adenine dinucleotide, NADH; lactate, LAC; ethanol, EtOH; formate; FMT; coenzyme A, CoA; fructose-1,6-bisphosphate, FBP; pyruvate, PYR; glucose-6-phosphate, G6P; glyceraldehyde-3-phosphate, GAP; dihydroxyacetone phosphate, DHAP; Phosphoenolpyruvate, PEP; 3-phopho-D-glycerate, G3P; fructose-6-phosphate, F6P; acetaldehyde, ACALD; acetylphosphate, ACPH; 2-Phospho-D-glycerate, G2P; 1,3-bisphosphoglycerate (Glycerate-1,3-bisphosphate), G13P2; acetolactate, ACLAC; acetyl-CoA, ACCoA; Glucose, GLC; hexokinase, HXK; phospho-glucose-isomerase, PGI; phosphofructo kinase, PFK; aldolase, ALD; acetolactate decarboxylase, ACLACD; triosephosphate isomerase, TPI; glyceraldehyde-3-phosphate dehydrogenase, GAPDH; phosphoglycerate kinase, PGK; phosphoglycerate mutase, PGM; enolase, ENO; lactate dehydrogenase, LDH; Acetolactate dehydrogenase, ACALDH; phosphotransacylase, PTA; acetate kinase, ACK; adenosine triphosphate hydrolase, ATPase; acetolactate synthase, ALS; D-beta-Hydroxybutyrate dehydrogenase, BDH; pyruvate kinase, PYK; phosphotransferase system, PTS; pyruvate dehydrogenase, PDH; alcohol dehydrogenases, ADH.
